# Pharmacokinetics of the Recalcitrant Drug Lamotrigine: Identification and Distribution of Metabolites in Cucumber Plants

**DOI:** 10.1021/acs.est.3c06685

**Published:** 2023-11-07

**Authors:** Moran Madmon, Yifat Zvuluni, Vered Mordehay, Ariel Hindi, Tomer Malchi, Eyal Drug, Moshe Shenker, Avi Weissberg, Benny Chefetz

**Affiliations:** † Department of Analytical Chemistry, Israel Institute for Biological Research, 7410001 Ness Ziona, Israel; ‡ Department of Soil and Water Sciences, Institute of Environmental Sciences, Faculty of Agriculture, Food and Environment, The Hebrew University of Jerusalem, 7610001 Jerusalem, Israel

**Keywords:** wastewater, chemical structure, irrigation, transformation, conjugation, lamictal

## Abstract

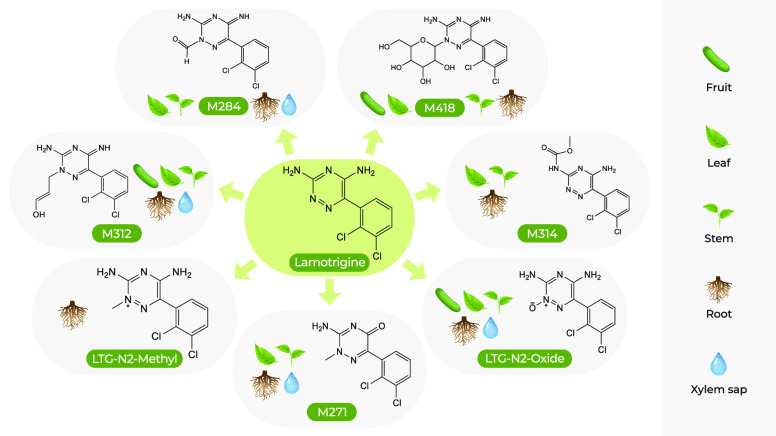

Treated wastewater is an important source of water for irrigation. As a result, irrigated crops are chronically exposed to wastewater-derived pharmaceuticals, such as the anticonvulsant drug lamotrigine. Lamotrigine is known to be taken up by plants, but its plant-derived metabolites and their distribution in different plant organs are unknown. This study aimed to detect and identify metabolites of lamotrigine in cucumber plants grown for 35 days in a hydroponic solution by using LC-MS/MS (Orbitrap) analysis. Our data showed that 96% of the lamotrigine taken up was metabolized. Sixteen metabolites possessing a lamotrigine core structure were detected. Reference standards confirmed two; five were tentatively identified, and nine molecular formulas were assigned. The data suggest that lamotrigine is metabolized via N-carbamylation, N-glucosidation, N-alkylation, N-formylation, N-oxidation, and amidine hydrolysis. The metabolites LTG-N_2_-oxide, M284, M312, and M370 were most likely produced in the roots and were translocated to the leaves. Metabolites M272, M312, M314, M354, M368, M370, and M418 were dominant in leaves. Only a few metabolites were detected in the fruits. With an increasing exposure time, lamotrigine leaf concentrations decreased because of continuous metabolism. Our data showed that the metabolism of lamotrigine in a plant is fast and that a majority of metabolites are concentrated in the roots and leaves.

## Abstract 2

Pharmacokinetics data are reported for the persistent drug lamotrigine in cucumbers that were exposed to lamotrigine for 35 days.

## Introduction

Many regions of the world experience water scarcity due to climate change, population growth, increasing demand for food, and urbanization.^1−3^ Thus, treated wastewater (TWW) has become an alternative water source for crop irrigation.^4^ The current quality guidelines for irrigation with TWW lack standards and/or limits for pharmaceutical and personal care products.^5−9^ Thus, using TWW for crop irrigation, pharmaceutical and personal care products and other contaminants of emerging concern are unintentionally introduced into agricultural soils and thereafter to the food chain^4,10^ and ultimately to consumers.^11−13^

Lamotrigine (LTG), a phenyl triazine-class anticonvulsant drug used for the treatment of epilepsy and bipolar disorder,^14,15^ is one of the most commonly found pharmaceuticals in TWW.^16,17^ It has been detected in various environmental niches contaminated with effluents in the United States.^16^ Similarly, Wood et al.^18^ reported LTG in aqueous environments across South Africa. Recently, Ben Mordechay et al.^19,20^ reported that LTG was detected in 94% of crop irrigation water samples (i.e., TWW) and exhibited a relatively high median concentration of 649 ng L^–1^. In an earlier study, Grossberger et al.^21^ reported that LTG is present in top soils irrigated with TWW in a concentration range of 2.2–4.4 ng g^–1^, which increased with the number of irrigation cycles.

The fate of pharmaceutical and personal care products and other contaminants of emerging concern in the water–soil–plant continuum has been recently investigated and reviewed.^22,23^ Several studies have shown that root uptake of pharmaceuticals occurs and that some of them tend to be translocated and accumulate in different plant organs.^24−27^ On the basis of its physicochemical properties, nonionic molecules such as LTG can easily cross root membranes, move to the xylem sap, translocate predominantly with the transpiration stream, and accumulate mostly in the leaves.^28^ Interestingly, carbamazepine and LTG have similar log *K*_ow_ values (2.45 and 2.57, respectively) but exhibited different leaf concentrations; carbamazepine’s concentration in leaves of cucumber plants was ∼5 times higher than that of LTG. In tomato plants, that of carbamazepine was ∼100 times higher than that of LTG. Goldstein et al.^26^ suggested that within plant compartments, LTG is ionized and thus can be trapped as an ion in the vacuoles or bind to the negatively charged cell walls resulting in a reduced level of translocation to the shoot. Another possible explanation for the differences between carbamazepine and LTG may be related to their *in planta* metabolism. To the best of our knowledge, no scientific papers have reported the metabolism of LTG in plants. In the human body, LTG is extensively metabolized to yield LTG-N_2_-glucuronide, LTG-N_5_-glucuronide, LTG-N_2_-oxide, and LTG-N_2_-methyl.^17^ Similar to pharmacokinetic processes in the human body as well as reports on the metabolism of LTG by fungi,^29^ it is hypothesized that in plants, LTG is metabolized primarily through oxidation, reduction, and conjugation processes.^27^

The goals of this study were to detect and identify metabolites of LTG in roots, leaves, stems, xylem sap, and fruits of cucumber plants and to monitor their fate during the growing period. Cucumber was selected as a fast-growing model plant that can provide commercial-size fruits within ∼30 days in a hydroponic system allowing analysis of all plant organs (roots, stems, leaves, and fruits). The generated pharmacokinetics data (used to describe the fate of exogenous xenobiotics in the plant based on the processes of absorption, distribution, metabolism, and accumulation that are analogous to pharmacokinetics in animals^27^) will allow a better understanding of LTG *in planta* processes.

## Materials and Methods

### Chemicals

LTG (>98%) and 2,3,4,6-tetra-*O*-acetyl-α-d-glucopyranosyl bromide (98.5%) were purchased from TCI (Zwijndrecht, Belgium). LTG-N_2_-oxide (>95%), LTG-^13^C3, and 5-desamino 5-oxo-2,5-dihydro LTG (OXO-LTG; 98%), 2-methyllamotrigine methanesulfonate (LTG-N_2_-methyl) were purchased from TRC. All solvents (acetone, acetonitrile, methanol, and water) were LC-MS grade (Biolab Co., Jerusalem, Israel). NaOH was purchased from Honeywall Fluka (Tokyo, Japan). The following reagents were purchased from Sigma-Aldrich (St. Louis, MO): methyl chloroformate (99%), potassium carbonate, allyl bromide (99%), propionyl chloride (98%), 2-(2-bromoethyl)-1,3-dioxolane (96%), iodomethane (99%), formamide (99%), and sodium methoxide (99%).

### Growing Conditions

Cucumber seeds (*Cucumis sativus*, Hazera Genetics Ltd.) were germinated in vermiculite moistened with a saturated CaSO_4_·2H_2_O solution for 7 days. Then, each seedling was transferred into 3.4 L plastic jars containing a continuously aerated nutrient solution. The nutrient solution contained macronutrients [K_2_SO_4_, 0.7 mM; KCl, 0.1 mM; Ca(NO_3_)_2_·4H_2_O, 2.0 mM; MgSO_4_, 0.5 mM; KH_2_PO_4_, 0.1 mM] and micronutrients [FeEDTA, 10 μM; MnSO_4_·H_2_O, 0.5 μM; ZnSO_4_·7H_2_O, 0.5 μM; CuSO_4_, 0.2 μM; (NH_4_)_6_Mo_7_O_24_·4H_2_O, 0.01 μM; H_3_BO_3_, 10 μM]. The pH of the nutrient solution was 5.7.

The plants were grown in a temperature-controlled growth chamber with a 16 h/8 h day/night cycle at day and night temperatures of 25 ± 1 and 20 ± 1 °C, respectively. A relative humidity of 60–70% was maintained during the day and 80–100% during the night. After being conditioned for 7 days, the plants were exposed either to 0 or 250 μg L^–1^ LTG in a nutrient solution. This concentration was low enough not to affect plant growth and high enough to detect metabolites. During the experiment, fresh nutrient solutions (with and without LTG) were replaced every 4 days. Overall, the plants were grown for 42 days, with conditioning for 7 days, followed by exposure to LTG for 35 days. Plants that were exposed for 18 days were used for metabolite identification. For the pharmacokinetic experiment, plants (five replicates) were harvested after 10, 15, 20, and 23 days (with and without LTG). To obtain fruits, we used plants that were exposed for 35 days. Plants exposed to LTG did not exhibit macroscopic differences from non-exposed plants (control).

### Sampling and Sample Preparation

Five plants were harvested at each sampling time. Plants were divided into roots, stems, leaves, and fruits. All plant parts were washed with deionized water, air-dried before weighing, frozen at −20 °C, and freeze-dried. The freeze-dried samples were ground into a fine powder and stored at −20 °C until extraction. LTG and its metabolites were extracted by agitating 100 mg of freeze-dried samples with 1 mL of methanol in a polypropylene tube (Corning). The tubes were vortexed for 30 s, sonicated (25 min at 25 °C), agitated (190 rpm for 50 min), and centrifuged (4000*g* for 5 min). The filtered supernatants were diluted at a 1:3 or 1:100 ratio with deionized water prior to high-resolution (HR) MS analysis. The recoveries of LTG from different plant organs were 100%, 78%, 86%, and 95% for roots, stems, leaves, and fruits, respectively.

Sampling of xylem sap was accomplished by using a diaphragm pump connected to a pressure regulator set to −0.8 bar. Plants were detopped below the first real leaf using a surgical blade. The detopped plants were fitted to a silicon connector connected to Teflon tubing linked to a 50 mL tube that was connected via an outlet to the manifold of the vacuum pump. The sap collected for the first 30 min was discarded because of a possible mixture with phloem sap. Xylem sap was subsequently collected over 90–120 min. Before analysis, each sample was spiked with 1 ng mL^–1^ LTG-^13^C3.

### Quantitation of LTG, LTG-N_2_-oxide, and LTG-N_2_-methyl

A sample (0.1 g dry weight) was placed in a 15 mL tube (Greiner Bio-One); then, 1 mL of MeOH was added, and the tubes were vortexed (30 s), agitated (200 rpm for 50 min), and centrifuged (4000*g* for 10 min). Subsequently, 80 μL of the supernatant was added to 10 μL of MeOH and spiked with 10 μL of 6 μg mL^–1^ LTG-^13^C3 for roots and 1.2 μg mL^–1^ for shoot samples. The mixture was diluted 1:100 and 1:20 for the root and shoot samples, respectively, with mobile phase A (0.2% formic acid in Milli-Q water) and filtered through a regenerated cellulose filter (0.22 μm; Phenomenex). LTG was quantified using an LC-MS system containing a Dionex Ultimate RS 3000 UPLC system coupled to a Q-Exactive Focus MS instrument equipped with a heated electrospray ionization source (Thermo Fisher Scientific Inc.). Chromatographic separation was performed by using a Kinetex EVO C18 column (2.1 mm × 100 mm, 2.6 μm; Phenomenex). The elution gradient was 10% mobile phase B (0.2% formic acid in ACN) for 5 min, followed by a linear gradient of mobile phase B to 90% for 0.5 min. The flow was held at 90% B for 2.5 min, switched back to 10% B, and held for an additional 8.5 min to equilibrate the column for the following analysis. The total LC cycle time was 17 min. The flow rate was 0.3 mL/min, and the column temperature was 40 °C. The injection volumes were 3 μL. Quantification of LTG-N_2_-oxide and LTG-N_2_-methyl was performed using the LC quadrupole linear ion trap (QTRAP) system, as detailed below.

### LC-HRMS (Orbitrap) for Detecting Metabolites

The HPLC–MS system consisted of an Agilent (Palo Alto, CA) 1290 high-performance LC system coupled to a Thermo Scientific Orbitrap MS instrument (Q-Exactive Plus; Thermo Fisher Scientific, Bremen, Germany) operated with an electrospray ionization source. Chromatographic separation was carried out using a reversed-phase column (Gemini C18, 3.0 μm, 150 mm, and 2.1 mm inside diameter, Phenomenex) at 40 °C with a flow rate of 0.3 mL min^–1^. The gradient program [solvent A, water with 5% MeOH; solvent B, MeOH (both containing 1 mM ammonium formate)] was as follows: 0 to 40 min, a linear increase from 0% to 95% B, a hold of 5 min at 95% B, followed by a fast return to 0% B, and a 10 min equilibration period. The Orbitrap operating parameters were as follows: electrospray voltage of 1.25 kV, sheath gas flow rate of 45 (arbitrary units), auxiliary gas flow rate of 10 (arbitrary units), sweep gas flow rate of 2 (arbitrary units), auxiliary gas heater temperature of 400 °C, and capillary temperature of 275 °C. The instrument was calibrated using a positive electrospray ionization calibration solution prepared according to the manufacturer’s instructions.

The Q-Exactive instrument was operated in full mass scan and data-dependent MS/MS (full MS/dd-MS^2^) in positive ion mode. The detection process was conducted in a data-dependent acquisition strategy in which the mass spectrometer selects and isolates the top 10 most intense precursor ions for fragmentation. The MS spectra were acquired in the range of *m*/*z* 50–750, and the mass resolution was set to 70 000. The automatic gain control target was set at 1 × 10^6^, with an injection time of 100 ms. The mass resolution of dd-MS^2^ in the data-dependent scan was set to 17 500. The automatic gain control target and maximum ion trap were set at 2 × 10^5^ and 50 ms, respectively. The collision energy was set as 45 eV. The mass resolution of the data-independent scan was set at 17 500.

### Structure Elucidation by Data Analysis

The full MS/dd-MS^2^ data files were imported into Compound Discoverer 3.0 (Thermo Fisher Scientific) for data processing. The workflow utilized for the data analysis is shown in Scheme S1. This method was based on a series of consecutive filters (Table S1). The raw data files were marked as samples or blanks to remove the existing background molecules. The alignment model was based on an adaptive curve with a maximum retention time shift of 0.5 min. The precursor ions were extracted according to the selected sodium, ammonium, and chloride adducts. In addition to lists of possible transformations that already existed in the software, we generated a plausible transformation list. The latter was based on the literature reported for LTG and related compounds. A comparison was performed between the detected parent ions and the expected parent ions, based on the mass tolerance set at 3 ppm, an intensity tolerance of 30%, a signal-to-noise threshold of 3, a minimum peak intensity of 50 000, and one isotope. Additionally, structure elucidation of the extracted masses and parent ions was achieved by a fragment ion searching scoring node. Scoring was used to predict in silico fragments (from MS^2^ experiments) based on the structures of the parent compounds. Using this method to reduce the amount of chemical information obtained by HRMS analysis leads to the acceptance of transformation products that necessarily belong to the parent LTG core structure.

### LC-QTRAP-MS Analysis to Improve the Detection Sensitivity of the LTG Metabolites

The analytes were separated using an Agilent 1290 high-performance LC system, which consisted of a model 1290 infinity binary pump containing a jet weaver V35 mixer, a model 1290 infinity autosampler, and a model 1290 infinity thermostat column compartment. Gradient elution was performed on a reverse-phase separation column (Gemini C18, 3.0 μM, 150 mm, 2.1 mm inside diameter, Phenomenex) with a flow of 0.3 mL min^–1^. The column was maintained at 25 °C. Mobile phase A consisted of 95% water and 5% MeOH, and mobile phase B consisted of MeOH, both containing 1 mM ammonium formate. The elution gradient mode was as follows: starting with 0% mobile phase B and ramped linearly to 95% B over 35 min. Then, the flow was held at 95% B for 5 min, switched back to 0% B, and held for an additional 10 min to equilibrate the column for the following analysis. The total LC cycle time was 50 min.

The metabolites of LTG in the plant were monitored using MS^2^ experiments with an Applied Biosystems 5500 QTRAP LIT quadrupole mass spectrometer (AB SCIEX, Foster City, CA) controlled by Analyst (version 1.6.2) and equipped with a Turbo V ion source operated with electrospray ionization (ESI) in positive ion mode. The ESI inlet conditions were as follows: gas 1, air (40 psi); gas 2, air (60 psi); ion spray voltage, 4500 V; ion source temperature, 600 °C; curtain gas, nitrogen (35 psi). For the enhanced product ion (EPI) (MS/MS) experiments, the collision gas was set at “high” and the collision energy was set between 10 and 80 eV. For each metabolite, transitions from the MH^+^ precursor ion to at least the two most dominant or indicative product ions were monitored. The declustering potential, entrance potential, and collision exit potential for all MRM transitions were 120, 10, and 13 V, respectively. The dwell time was 10 ms. The details of the MRM transitions, collision energy, and retention time are listed in Table S2. The mass spectral data for all LTG metabolites are presented in Figures S1–S16.

### Synthesis of the LTG Metabolites (M271, M284, M312, M314, and M418)

Structural identification of the LTG metabolites was performed with the aid of an “in-house” organic synthesis of chemical standards (some of them required two-step sequential reactions). The crude reaction mixture containing the synthesized standards was diluted 1:100 or 1:1000 with water before HRMS analysis. A mass spectral comparison (MS/MS fragmentation) between each suspected metabolite and the in-house-synthesized chemical standard was performed. All of the synthetic schemes are illustrated in Schemes S2–S6. The synthesis routes are as follows. For M271, potassium carbonate (1 mg) and iodomethane (2 μL) were added to 1 mL of 5 μg mL^–1^ LTG, in ACN. The reaction mixture was then stirred and heated to 50 °C for 30 min. For M284, 2 μL of a 0.5 M sodium methoxide in a methanol solution was added to 1 mL of 10 μg mL^–1^ LTG in formamide. The reaction mixture was heated to 70 °C and stirred for 5 h. For M312, 1 mg of potassium carbonate followed by 2 μL of bromomethyl dioxolane was added to 1 mL of 200 μg mL^–1^ LTG in ACN. The reaction mixture was stirred and heated to 55 °C for 2 h. Then, 100 μL of the reaction mixture was diluted 1:1 with water, and 1 μL of a 32% HCl solution was added. The diluted solution was stirred and heated to 55 °C for 1 h. For M314, 10 mg of potassium carbonate followed by 20 μL of methyl chloroformate was added to 200 μL of 4 μg mL^–1^ LTG in ACN. The reaction mixture was then stirred at room temperature for 2 h. For M418, 1 mg of 2,3,4,6-tetra-*O*-acetyl-α-d-glucopyranosyl bromide was added to 200 μL of 1 mg mL^–1^ LTG in methanol. The reaction solution was stirred and heated to 55 °C for 2 h. Subsequently, 1 μL of a 1 M sodium hydroxide solution was added to 100 μL of the reaction solution. The solution was then stirred and heated to 40 °C for 50 min.

The identification methodology was established by the conducting specific data-independent acquisition MS/MS (DIA-MS^2^) methods for each metabolite as well as gathering all of the mass spectral information and profound mass spectrometry interpretation of the dissociation processes. Notably, the interpretation of all mass spectra was performed manually, utilizing our comprehensive data set of ESI-MS-MS fragmentation rules. Plausible structures were proposed for each metabolite as detailed below. When a commercial standard was not available (for M271, M284, M312, M314, and M418), an in-house synthesis of the standards was conducted. Metabolites were confirmed by matching the retention times and mass spectral data of the observed metabolites in the plant extracts to standards (a commercially available or in-house-synthesized standard). An exact match between the LC and MS data increases the level of confidence of identification.

### Statistical Analysis

Statistical analysis (nonparametric Turky–Kramer HSD test; *P* < 0.05) was performed using JMP Pro 13 (JMP, version 13; SAS Institute Inc., Cary, NC).

## Results and Discussion

The uptake and accumulation of LTG by plants have been previously reported.^22,30^ However, all studies were focused on the parent compound (i.e., LTG); no information regarding *in planta* metabolism or the identification of LTG metabolites has been reported. This complementary information is needed for a comprehensive assessment of plant pharmacokinetics (uptake, translocation, and metabolism of pharmaceuticals) of an important contaminant of emerging concern, LTG. Moreover, this information is needed to better assess the health risks associated with the consumption of produce containing LTG conjugates.

### Detection and Identification of LTG Metabolites

Our mass-balance data showed that <5% of the taken-up LTG (i.e., LTG removed from the nutrient solution) was detected in the plant as the parent compound. Because LTG metabolites were not detected in nutrient solutions, we suggest that 96.4 ± 0.8% of the taken-up LTG was metabolized. This highlights the importance of the detection and identification of LTG metabolites.

One of the major challenges in this research was the peak digging process (i.e., peaks observed in the LTG-exposed plants that were absent in the non-exposed plants) and finding the single correct molecular modification that fits the LC and MS parameters, such as the accurate mass (±3 ppm), fragmentation patterns, retention time, and ^13^C:^37^Cl isotopic ratio (±30% of the expected relative intensity). LC-HRMS libraries are still limited, and even if they exist, the comparison between ESI-MS/MS spectra acquired from different instrument types is not a straightforward task.^31^ Weissberg et al.^32^ constructed a comprehensive empirical set of ESI-MS/MS fragmentation rules, based on a systematic study. The developed methodology was applied to the identification of “unknown” small organic compounds by matching an empirical ESI-MS/MS spectrum to the ESI-MS/MS spectrum of a predicted chemical structure. Schymanski et al.^33,34^ characterized five identification confidence levels in HRMS analysis, which rank the quality of identification according to the study data. The molecular formulas/modifications that met the detection criteria (LC- and MS-defined parameters are listed in Table S1) and the levels of confidence of the metabolites are listed in Table 1.

**Table 1:**
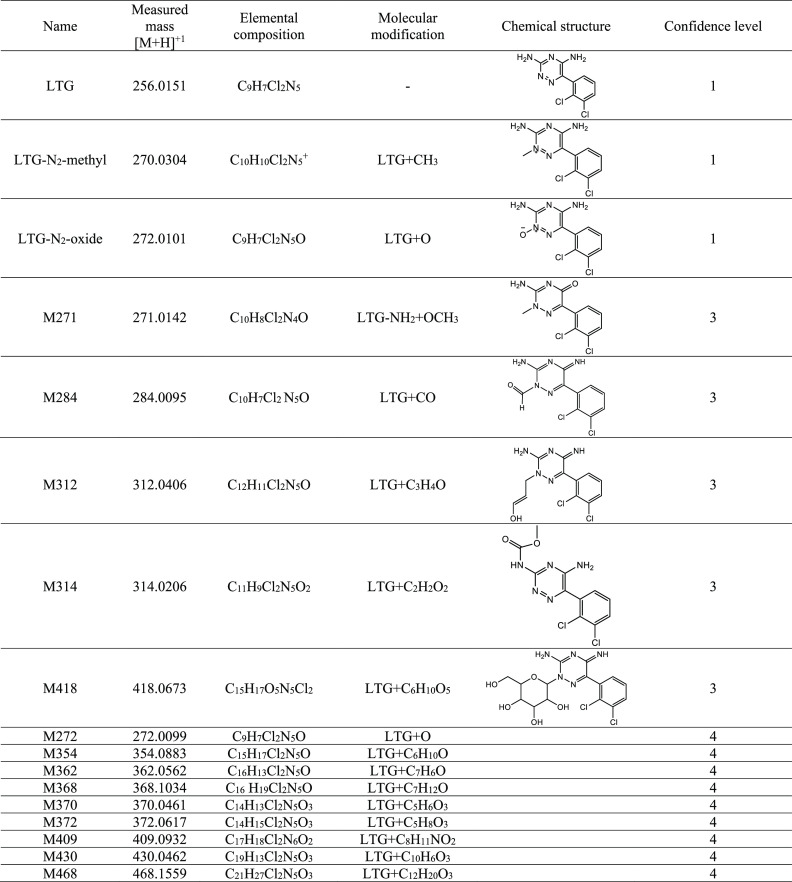
Lamotrigine (LTG) and Its Metabolites Identified in Roots, Stems, and Leaves of Cucumber Plants Exposed to LTG for 10, 15, 20, and 23 Days^a^

a Measured masses, elemental compositions, molecular modifications, and tentative chemical structures based on confidence level^32,33^ are presented.

In this study, seven metabolites (Table 1) exhibited MS/MS data that were sufficient to propose possible structures based on our comprehensive empirical set of ESI-MS/MS fragmentation rules.^32^ For two of the proposed metabolites, LTG-N_2_-methyland LTG-N_2_-oxide, commercial standards were available, and their structures were confirmed by retention times and fragmentation pattern matching [i.e., confidence level 1 (Figures S1 and S3, respectively)]. Other proposed metabolites lack authentic standards; the level of identification confidence was 3 (Figures S2 and S4–S7).^33^ For the nine other metabolites, only molecular formulas were unambiguously assigned [confidence level 4 (Figures S8–S16)].

Five of the observed metabolites (M272, M312, M314, M370, and M372) were previously reported in another biological system; however, plausible structures were not proposed.^29^ LTG-N_2_-oxide was also observed as a transformation product in an abiotic system where LTG was transformed by the manganese dioxide mineral, birnessite.^35^ In this study, we highlight the vast conjugation as the major transformation pathway. Of the 16 metabolites detected, 11 were suggested to be LTG conjugates. Conjugates attached to the dichlorobenzene ring or transformation over the dichlorobenzene ring were not detected, suggesting the low reactivity of the benzene ring for biotic metabolism and the high reactivity of the triazine structure (1,2,4-triazine) for metabolism. This is analogous to many studies reporting the fate and metabolism of commonly used triazine-based herbicides such as Atrazine, Simazine, and Metribuzin.^36−39^ LTG possesses more than one nucleophilic site in the 1,2,4-triazine moiety; therefore, the exact location of the substituents could not be determined. However, the N_2_ position is the most favorable location as reported in many studies of LTG metabolites, indicating the enhanced basicity of nitrogen at the N_2_ position. Therefore, most of the identified metabolites were assigned to their substituents at the N_2_ posision of the triazine ring.

### Pharmacokinetics

**Figure 1:**
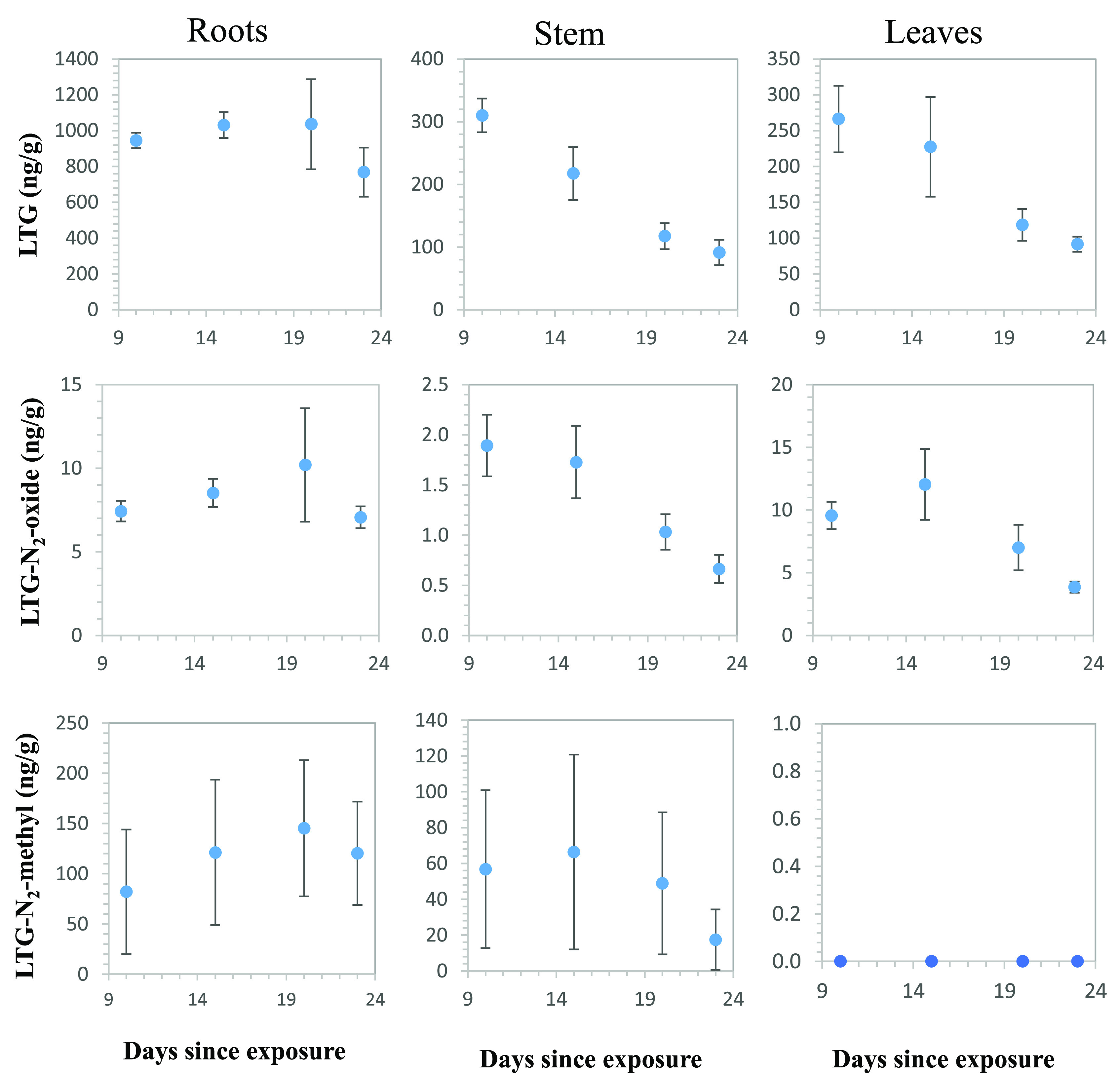
Concentrations of lamotrigine (LTG), LTG-N_2_-oxide, and LTG-N_2_-methyl in roots, stems, and leaves of cucumber plants (five replicates) exposed to LTG for 10, 15, 20, and 23 days. Average data for five replicates are presented; bars represent standard errors.

This section describes the distribution profiles of LTG and its 16 metabolites detected in the shoots and roots of cucumber plants during the growing period (i.e., plants exposed to LTG for 10, 15, 20, and 23 days). For LTG and the two derivatives, LTG-N_2_-oxide and LTG-N_2_-methyl, identification was performed via comparison with commercial standards (confidence level 1); therefore, quantification was performed, as summarized in Figure 1. The distribution profiles of all of the other metabolites with confidence levels of 3 or 4 are presented in the Supporting Information and are described in a semiquantitative manner.

The LTG concentration in the roots did not change over time with an average concentration of 945 ng g^–1^. However, the concentration of LTG in the stems and leaves decreased with exposure time. In both organs, the concentration of LTG decreased from ∼300 to 90 ng g^–1^. Goldstein et al.^26^ suggested that LTG is taken up in its neutral form; however, within the cytosol, LTG is positively charged (due to pH differences) and thus trapped as an ion in the vacuoles or bound to the negatively charged cell walls. This resulted in a reduced level of translocation of the shoot. Most likely, the constant concentration of LTG in the root is the result of a steady-state condition that balances uptake, translocation, and metabolism. The fraction that is translocated to the shoots is metabolized at a rate faster than the rate of introduction, resulting in a decreased concentration of LTG over time. Therefore, uptake models based on parent compound data (e.g., LTG) underestimate the actual plant uptake.

The concentration of the metabolite LTG-N_2_-oxide was significantly lower than that of LTG, suggesting that it is a short-lived metabolite or N oxidation is not a major mechanism of transformation. Similar to the case for LTG, its concentration in the shoots decreased with time; its concentration in the stem was lower than that in the leaves. The metabolite LTG-N_2_-methyl was not detected in the leaves. Its average concentration in the root was 117 ng g^–1^ and in the stems was 47 ng g^–1^. However, it showed high variability, making it difficult to reach conclusions about its fate and kinetics.

The root:leaf concentration ratios for LTG increased with exposure time (3.5, 4.5, 8.7, and 8.4 for exposure times of 10, 15, 20, and 23 days, respectively). For LTG-N_2_-oxide, the values of these concentration ratios were much smaller but exhibited a similar trend; the corresponding ratios increased with time (0.8, 0.7, 1.5, and 1.8, respectively). These data further highlight the importance of leaf transformation. When the concentration ratios were normalized to the dry weight of each organ (Table 2), the corresponding ratios for LTG were on average 1, with no significant change over time. Similarly, for LTG-N_2_-oxide, the weight-normalized corresponding ratios did not change with time, with an average value of 0.2. The observed trends were due to the increasing mass of the leaves compared to the mass of the roots over the growing period.

**Table 2:** Lamotrigine (LTG) and Its Metabolites in Roots, Stems, and Leaves of Cucumber Plants Exposed to LTG for 10, 15, 20, and 23 Days^a^

	day 10			day 15			day 20			day 23		
	roots	stem	leaves	roots	stem	leaves	roots	stem	leaves	roots	stem	leaves
LTG	47.2	12.3	40.4	32.7	13.4	53.9	49.1	11.2	39.7	44.5	11.9	43.6
LTG-N_2_-methyl	96.3	3.7	0	92.9	7.1	0	100.0	0	0	100.0	0	0
LTG-N_2_-oxide	16.3	7.5	76.1	8.3	8.0	83.8	14.8	6.8	78.4	15.8	7.5	76.7
M271	51.1	12.0	36.9	37.4	14.3	48.3	50.3	8.0	41.7	47.1	6.4	46.5
M272	17.6	11.9	70.5	9.7	13.3	77.0	5.9	2.5	91.6	24.4	8.8	66.7
M284	76.0	4.2	19.8	71.3	4.4	24.4	64.3	4.1	31.7	49.7	3.5	46.8
M312	39.5	7.0	53.5	21.8	12.6	65.6	29.3	11.6	59.1	31.0	12.1	56.9
M314	15.4	5.6	79.0	2.9	1.5	95.6	17.4	4.0	78.6	15.3	4.4	80.3
M354	5.9	2.3	91.8	2.5	4.9	92.6	9.6	3.3	87.1	14.2	3.6	82.2
M362	99.9	<1	0	99.2	<1	0	99.9	<1	0	99.8	<1	0
M368	6.5	1.8	91.7	4.2	3.9	91.9	10.9	2.7	86.5	13.6	2.8	83.6
M370	7.2	2.3	90.5	2.6	2.1	95.3	3.1	<1	96.2	2.7	1.2	96.1
M372	35.6	64.4	0	43.2	56.8	0	100.0	0	0	100.0	0	0
M409	32.4	67.6	0	23.3	76.7	0	53.5	46.5	0	45.4	54.6	0
M418	5.9	1.6	92.5	5.5	3.7	90.8	3.2	1.8	95.0	2.7	1.5	95.8
M430	100.0	0	0	100.0	0	0	100.0	0	0	100.0	0	0
M468	56.1	6.1	37.8	32.4	8.6	59.0	81.1	3.6	15.4	69.4	4.5	26.0

a Data for the peak area normalized to the dry weight of each organ (peak area × organ weight/plant weight) are presented. Data are averages of five replicates. For metabolites M370 and M409, multiple isomers were obtained, and aggregated data are shown.

For the metabolites with confidence levels of 3 and 4 (Table 1), all were detected in the roots, 13 in the stem, 10 in leaves, and 8 in fruits. The metabolites M284, M362, M430, and M468 were dominant in roots (Table 2). The leaf-dominant metabolites were M272, M312, M314, M354, M368, M370, and M418. M362, M372, M409, and M430 were not observed in the leaves. All metabolites exhibited higher intensity (peak area) in the roots than in the stems throughout the exposure period (exposure for 10, 15, 20, and 23 days). In the leaves, the metabolites M354, M368, M370, and M418 exhibited peak areas larger than those in the roots. This trend was maintained throughout the exposure period.

To evaluate the proportion of each metabolite within different plant organs over the exposure period, we normalized the peak area of each metabolite to the dry weight of each organ (Table 2). Although the stem weight was greater than the root weight, the normalized intensities of the metabolites in the stem were lower than those in the roots (except for M409), suggesting that metabolites were formed and/or accumulated in the roots rather than in the stem. The differences in weight normalization (i.e., relative concentration) of the metabolites highlighted the fact that the roots and leaves are most likely the organs for LTG metabolism and/or accumulation, while the stems function as a translocation organ.

For most of the metabolites, the normalized peak areas in the roots, stems, and leaves showed no significant changes with exposure time (Figures S17–S30). However, for M354 (Figure S22) while the normalized peak area showed no significant trend with exposure time in the roots, a significant decrease in peak area for both the stem and leaves was observed. For the stem, the normalized peak area decreased from 143 to 44 and for the leaves from 2700 to 490 for exposure days 10 and 23, respectively. A similar trend was observed for M368 (Figure S24), and the normalized peak area was constant in the roots but significantly decreased in the peak area for both the stem and leaves. For the stem, the normalized peak area decreased from 12 to 3 and for the leaves from 284 to 43 for exposure days 10 and 23, respectively. M372 (Figure S26) exhibited a normalized peak area of 2.5 in the stem during an exposure time of 10 days; on days 20 and 23, it was absent. M468 (Figure S30) exhibited a decreasing trend in the leaves from 18 to 2.6 during the exposure time. We suggest that the metabolites for which their relative concentrations decreased with an increase in exposure time or with an increase in growing time are subject to additional metabolism or translocated to the roots.

To reveal where metabolites are formed, xylem sap, which carries nutrients and water from the root system to the leaves, was analyzed at different time points along the exposure period. The following metabolites were detected in xylem sap: LTG-N_2_-oxide, M284, M312, M354, and M370. The relative concentrations of these metabolites did not change during exposure (Figure S31). All other metabolites that were not detected in the xylem sap were therefore produced in leaves and reached the roots via the phloem sap, which carries photosynthesis products to all of the other plant organs. Alternatively, these metabolites could have been produced in the roots, but they were immobile and confined to the root system. LTG-N_2_-oxide, M284, M312, M354, and M370 were most likely produced in roots and translocated to leaves. It is interesting to note that the leaf:root ratios of LTG-N_2_-oxide, M284, and M312 were ∼1 and did not change with exposure time. However, for M354, the leaf:root ratios decreased from 23.4 to 2.8 during the exposure time, and for M370, the ratio varied from 10 to 5.9 with no significant change over time. These data suggest that LTG-N_2_-oxide, M284, and M312 were further metabolized in the leaves. This was also the case for M354, which exhibited accumulation and subsequent decomposition. M370 accumulated in the leaves over time, and its proportion in the leaves increased to 90–96% with time (Table 2).

To obtain fruits, the plants were grown for an additional 2 weeks. LTG and LTG-N_2_-oxide concentrations in the fruits were 295 and 10 ng g^–1^, respectively. LTG-N_2_-methyl was not detected in the fruits. The fruit’s LTG concentration is only ∼30% of its concentration in the root. This supports the hypothesis that the translocation of LTG to above-ground organs is limited and that the level of LTG in fruits in general is expected to be low.^19,20,40^ The following metabolites were detected in the fruits: M272, M312, M354, M368, M370, M418, and M468. Compared to those in leaves, the intensities of the metabolites in the fruits are in most cases negligible. M418, a sugar-containing metabolite that was not detected in the xylem sap, exhibited a relatively high normalized peak area intensity in the fruit, suggesting that this metabolite is most likely formed in the leaves and translocated to the fruit via the phloem sap.

The process of plant metabolism aims to increase the compound’s hydrophilicity through activation and conjugation, thus weakening its ability to solubilize in the phospholipid bilayer membrane and its ability to undergo cell-to-cell diffusion.^41,42^ This process is best described by the green liver model that divides plant metabolism into three or four phases.^42^ In phase I metabolism, the initial properties of a parent compound are transformed through oxidation, reduction, or hydrolysis to increase the polarity and, thus, the solubility of the compound. Of the compounds identified in this study, LTG-N_2_-oxide is a classic example of phase I metabolism, oxidation of LTG. Phase II consists of conjugation of the parent compound or its metabolite with a sugar, glutathione, or amino acid to increase its hydrophilicity. The conjugation results in a lower biological activity and a higher water solubility in comparison to those of the parent compound. Metabolite M418 is a classic example of an addition of glucoside to the parent compound. A similar metabolic pathway was found in sugar beet for the 1,2,4-triazine herbicide Metamitron.^43^ The other two phase II metabolites, LTG-N_2_-methyl and M271, are nonclassic examples, which undergo a methylation and amidine hydrolysis along with methylation, respectively. This process is common in the biosynthesis of endogenous plant compounds and can occur with xenobiotics. The addition of the methyl group modulates the bioavailability, bioactivity, and reactivity of the acceptor molecule and thus part of the plant detoxification process.^44^ Phase III reactions are the elimination step, whereas compounds are sequestered out of the cytosol into the intracellular space, most commonly the vacuole where they are confined to prevent interactions in biochemical processes. Phase III may also include the formation of secondary conjugation such as with malonyl, which provides a signal for transport from the cytoplasm to the vacuole.^41^ There is evidence for several triazines that plants form secondary conjugation with Malonyl.^45^ This may be the process in some of the larger metabolites detected and listed in Table 1. Phase IV includes transformation reactions in which the compound may undergo further substitution, degradation, and/or partial utilization of the compound. This may include the conjugation of a xenobiotic with proteins, lignin, hemicelluloses, and pectin.^46^ At this stage, the compound is often termed a bound residue, and there is a lack of understanding regarding this process.

Two metabolites identified in this study do not fit into the plant metabolism model presented above: the addition C_3_H_4_O in M312 and the addition of C_2_H_2_O_2_ in M314. Interestingly, these metabolites were also detected in white-rot fungus.^29^ In a study on the metabolism of Diazinon, conjugates similar to these metabolites were found.^47,48^ However, these types of conjugates are generally missing from metabolic models. Moreover, most observed biotransformation reactions of LTG have not been previously reported in triazine herbicides.

## Environmental Implications

The insights gained in this work will aid in assessing *in planta* processes, i.e., pharmacokinetics data, as well as provide reliable measurements and modeling of uptake needed to assess exposure to LTG and other pollutants of emerging concern due to intensive irrigation of crops with TWW. Another implication to be considered is the environmental stability of the observed metabolites considering their possible decomposition to the intact LTG. Although it is possible that studies based on hydroponic culture may not fully reflect uptake processes in plants growing in soil, hydroponic exposure experiments are an ideal basis for studying the fate of pollutants in plants grown in the agricultural environment. Future complementary studies of the metabolite’s distribution and their quantitative determination in plant organs are still needed.

## Supporting Information Available

Detailed information about the detection process (Table S1 and Scheme S1), LC-MS/MS (MRM) parameters (Table S2), chemical reaction conditions (Schemes S2–S6), summaries of the identification process for each metabolite (Figures S1–S16), and distributions of metabolites in different plant organs (Figures S17–S30) and in xylem sap (Figure S31) (PDF)

## Dedication

The authors dedicate this paper to the memory of Professor Joel A. Pedersen (1965–2022), an inspiring colleague and a friend.
