# Supplementary material for: Pharmacokinetics of the Recalcitrant Drug Lamotrigine: Identification and Distribution of Metabolites in Cucumber Plants
Source: Environ Sci Technol. 2023 Nov 7;57(48):20228–37. doi: 10.1021/acs.est.3c06685 (PMC11137871; doi:10.1021/acs.est.3c06685)
Supplement: Supplementary file 1 — es3c06685_si_001.pdf [file es3c06685_si_001.pdf]

Supporting Information

Pharmacokinetics of the recalcitrant drug lamotrigine: Identification and distribution of metabolites in cucumber plants

Moran Madmon[‡†], Yifat Zvuluni[‡], Vered Mordehay[‡], Ariel Hindi[‡], Tomer Malchi[‡], Eyal Drug[†], Moshe Shenker[‡], Avi Weissberg[†]* and Benny Chefetz[‡]*

[†]Department of Analytical Chemistry, Israel Institute for Biological Research, Ness Ziona, Israel

[‡]Department of Soil and Water Sciences, Institute of Environmental Sciences, Faculty of Agriculture, Food and Environment, The Hebrew University of Jerusalem, Israel

***Corresponding authors**

E-mail: aviwe@iibr.gov.il

E-mail: benny.chefetz@mail.huji.ac.il

This supporting information contains 6 schemes, 2 tables and 31 figures.

Contents

Table S1. Settings for Compound Discoverer 3.0 nodes used in this study. 4

Table S2. Multiple reaction monitoring (MRM) conditions used to analyze lamotrigine and its metabolites. 5

Scheme S1: Compound Discoverer workflow for detection of lamotrigine metabolites in cucumber plants...7

Scheme S2: The reaction scheme and conditions for the synthesis of the M271.................8

Scheme S3: The reaction scheme and conditions for the synthesis of the M284.................8

Scheme S4: The reaction scheme and conditions for the synthesis of the M312.................9

Scheme S5: The reaction scheme and conditions for the synthesis of the M314.................9

Scheme S6: The reaction scheme and conditions for the synthesis of the M418...............10

Figure S1. Identification of LTG-N_2-methyl...12

Figure S2. Identification of M271...14

Figure S3. Identification of LTG-N_2-oxide...16

Figure S4. Identification of M284...18

Figure S5. Identification of M312...21

Figure S6. Identification of M314..24

Figure S7. Identification of M418..26

Figure S8. Detection of M272...27

Figure S9. Detection of M354...27

Figure S10. Detection of M362...28

Figure S11. Detection of M368...28

Figure S12. Detection of M370...29

Figure S13. Detection of M372...30

Figure S14. Detection of M409...30

Figure S15. Detection of M430...31

Figure S16. Detection of M468...31

Figure S17. Distribution profile of M271 32

Figure S18. Distribution profile of M272. 32

Figure S19. Distribution profile of M284. 32

Figure S20. Distribution profile of M312. 33

Figure S21. Distribution profile of M314. 33

Figure S22. Distribution profile of M354. 33

Figure S23. Distribution profile of M362. 34

Figure S24. Distribution profile of M368. 34
Figure S25. Distribution profile of M370. 34
Figure S26. Distribution profile of M372. 35
Figure S27. Distribution profile of M409. 35
Figure S28. Distribution profile of M418. 35
Figure S29. Distribution profile of M430. 36
Figure S30. Distribution profile of M468. 36
Figure S31. LTG-metabolites that detected in xylem sap during the exposure period. 36
References 37

Table S1. Settings for Compound Discoverer 3.0 nodes used in this study.

Node	Explication	Parameters
Input Files	LC/MS data file input	-
Select Spectra	Selects and retrieves the spectra for further processing	Min. Precursor Mass: 50Da<br>Max. Precursor Mass: 750Da<br>S/N Threshold: 3
Align Retention Time	Align Retention Time node compensates for small differences in the retention times of the components in the sequence of the sample runs.	Alignment Model: Adaptive curve<br>Max. shift: 0.5 min<br>Mass Tolerance: 3 ppm
Generate Expected Compounds	Generates expected compounds using single-step transformation and dealkylation tool.	Compound: LTG ($C_9H_7Cl_2N_5$) and LTG isomers.<br>Apply Dealkylation: True<br>Apply Dearylation: True<br>Transformation: Phase I, II<br>Max. all steps: 5<br>Ionization: [2M+H]+1, [2M+Na]+1, [M+H]+1, [M+Na]+1
Find Expected Compounds	Detects expected compounds in mass spectra	Mass Tolerance: 3 ppm<br>Intensity Tolerance: 30 %<br>Intensity Threshold: 0.1 %<br>Min. # isotopes: 1<br>Min. Peak Intensity: 50000
Detect Compounds	Detects compounds in the data using Compound Elucidator algorithm	Mass Tolerance: 5 ppm<br>Intensity Tolerance: 30 %<br>S/N Threshold: 3<br>Min. Peak Intensity: 10000<br>Ion: [M+H]+1, [M+K]+1, [M+Na]+1, [M-H]-1<br>Max. Element Counts: $C_{90}H_{190}Br_3Cl_4K_2N_{10}Na_2O_{18}P_3S_5$
Group Expected Compounds	Groups all detected expected compounds per file	R.T Tolerance: 0.3 min
Merge Features	Merges all detected features and provides the links for available explanation	Mass Tolerance: 3 ppm<br>R.T Tolerance: 0.3 min
Mark Background Compounds	Annotates and filters background compounds	Max. Sample/Blank: 3<br>Hide Background: True
FISH Scoring	Provides a score for detected expected compounds and annotates related MSn scans by fragment ion search	High Acc. Mass Tolerance: 3 ppm<br>Low Acc. Mass Tolerance: 5 ppm<br>Min. Fragment: *m/z* 50

Table S2. Multiple reaction monitoring (MRM) conditions used to analyze lamotrigine and its metabolites.

	Q1 mass (Da)	Q3 mass (Da)	Collision Energy (eV)	MRM transition ratio	R.T (min)
Lamotrigine	256.0	211.0	40.0	1.6	15.5
	256.0	109.0	70.0	1.4	
	256.0	145.0	50.0	1.4	
	256.0	157.0	50.0	1	
	256.0	159.0	40.0	1.1	
	256.0	187.0	40.0	1	
Lamotrigine-$^{13}C3$	259.0	214.0	40.0	3.9	15.5
	259.0	188.0	30.0	1	
	259.0	158.0	50.0	1	
	259.0	145.0	50.0	1.5	
LTG-N_2-Methyl	270.0	234.0	40.0	1	13.2
	270.0	201.0	40.0	5	
	270.0	185.0	50.0	1.8	
	270.0	172.0	50.0	2	
M271	271.0	243.0	30.0	3.2	23.7
	271.0	226.0	30.0	1	
	271.0	187.0	40.0	1.2	
	271.0	152.0	40.0	1.3	
LTG-N_2-oxide	272.0	242.0	30.0	9.3	13.9
	272.0	185.0	40.0	4.5	
	272.0	255.0	30.0	1	
	272.0	165.0	40.0	2.8	
	272.0	207.0	30.0	1.7	
M272	272.0	236.0	30.0	1.8	12.2
	272.0	227.0	40.0	1	
	272.0	188.0	40.0	2.1	
M284	284.0	256.0	30.0	19.7	18.3
	284.0	211.0	40.0	2.5	
	284.0	159.0	50.0	1	
M312	312.0	294.0	40.0	14.1	13.3
	312.0	256.0	40.0	15.1	
	312.0	172.0	50.0	2.8	
	312.0	211.0	50.0	1	
M314	314.0	282.0	30.0	32	18.9
	314.0	246.0	40.0	1	
	314.0	255.0	50.0	1.8	
	314.0	172.0	50.0	6.4	
M354	354.1	336.0	40.0	12.6	20.6
	354.1	256.0	40.0	25.3	
	354.1	310.0	40.0	1.2	
	354.1	268.0	40.0	1	
	354.1	211.0	60.0	2.5	

M362	362.1	256.0	30.0	24	18.1
	362.1	211.0	60.0	1	
	362.1	159.0	60.0	1	
M368	368.1	336.0	30.0	20	23.1
	368.1	256.0	40.0	2.6	
	368.1	172.0	50.0	7.3	
	368.1	310.0	40.0	1	
	368.1	298.0	40.0	2	
M370	370.0	256.0	40.0	2	16.0
	370.0	352.0	30.0	1	
	370.0	294.0	40.0	20	
	370.0	256.0	40.0	28.8	17.2
	370.0	352.0	30.0	1	
	370.0	324.0	40.0	8	
M372	372.0	354.0	30.0	0.5	19.2
	372.0	340.0	30.0	1.5	
	372.0	322.0	30.0	1	
	372.0	294.0	40.0	2	
	372.0	256.0	40.0	0.5	
M409	409.0	365.0	10.0	1	22.0
	409.0	256.0	20.0	3.4	
	409.0	110.0	20.0	19.3	
	409.0	365.0	10.0	1	23.0
	409.0	256.0	20.0	4	
	409.0	110.0	20.0	19.5	
M418	418.1	256.0	40.0	160	8.8
	418.1	211.0	50.0	3	
	418.1	187.0	50.0	1	
	418.1	166.0	50.0	2.8	
	418.1	159.0	60.0	4	
M430	430.0	415.0	40.0	20	22.1
	430.0	387.0	50.0	1	
	430.0	256.0	40.0	8.2	
	430.0	244.0	50.0	9.8	
	430.0	175.0	50.0	29.2	
M468	468.1	450.0	40.0	4.3	24.6
	468.1	432.0	50.0	1.4	
	468.1	256.0	50.0	21.4	
	468.1	211.0	60.0	1	

Schemes

This workflow finds related degradants predicted by the "generate expected compounds" node. Fragments of expected compounds are explained by the "FISH scoring" node. "Mark background compounds" node automatically hides compounds from blank files.

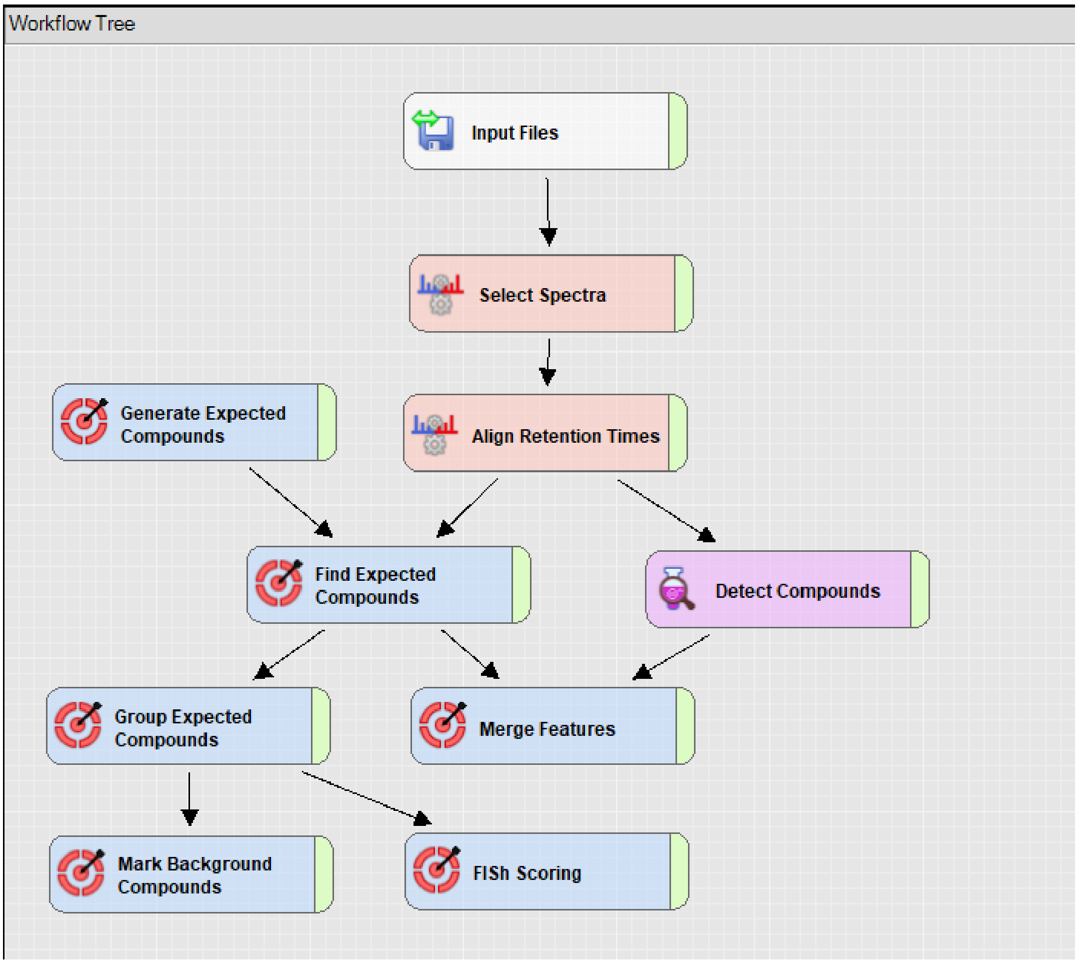

Scheme S1: Compound Discoverer workflow for detection of lamotrigine metabolites in cucumber plants.

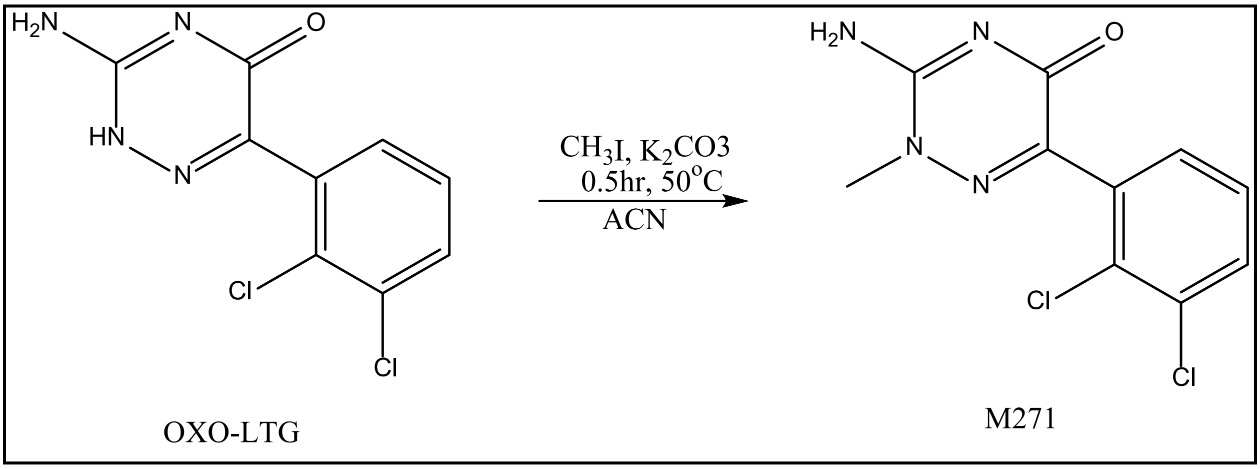

Scheme S2: The reaction scheme and conditions for the synthesis of the M271.

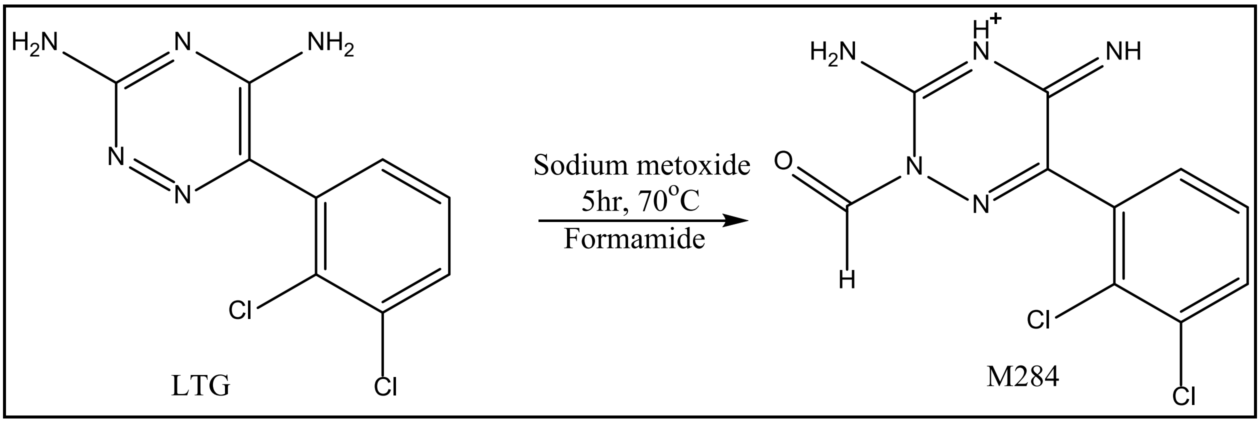

Scheme S3: The reaction scheme and conditions for the synthesis of the M284.

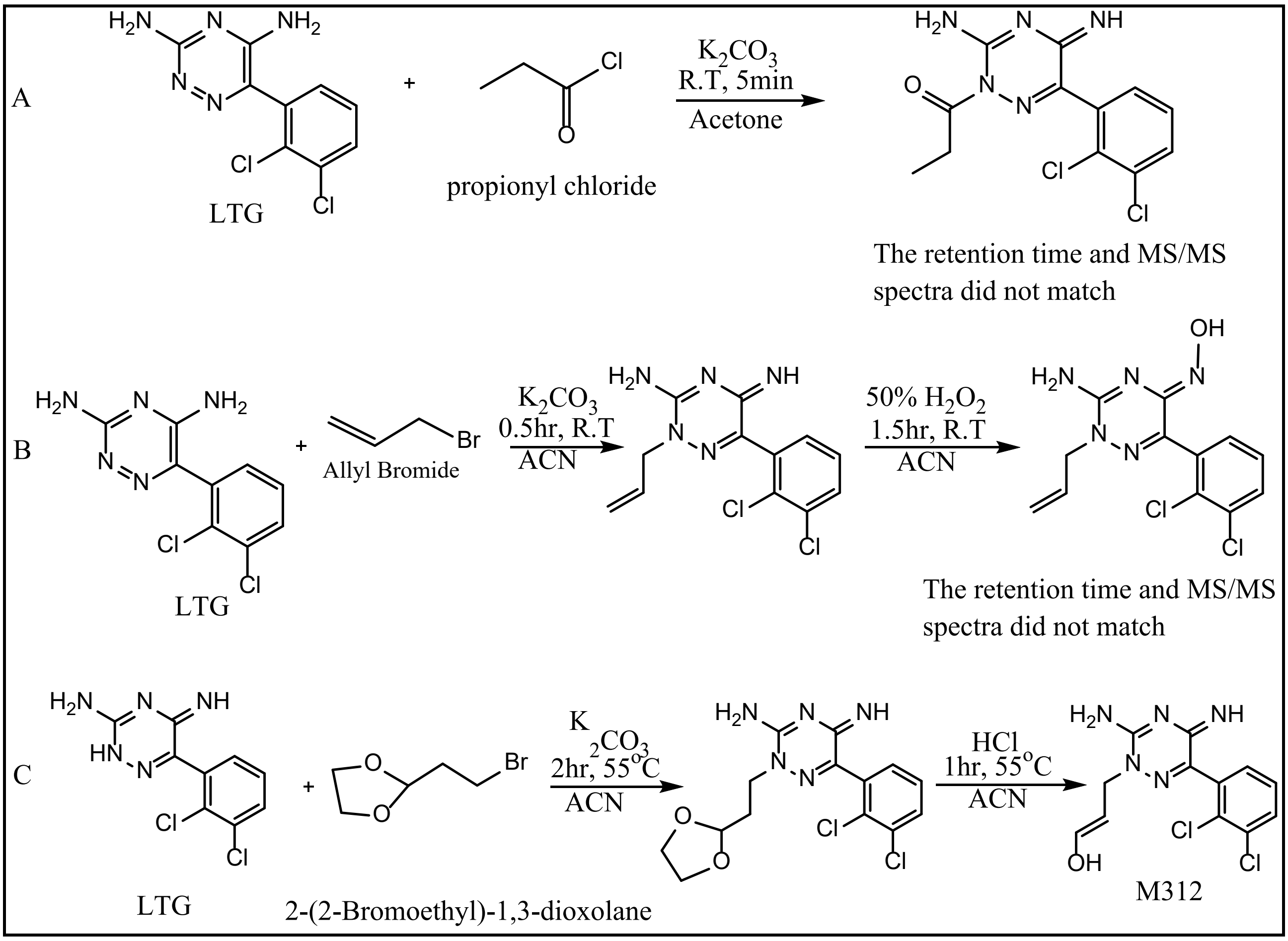

Scheme S4: The reaction scheme and conditions for the synthesis of the M312.

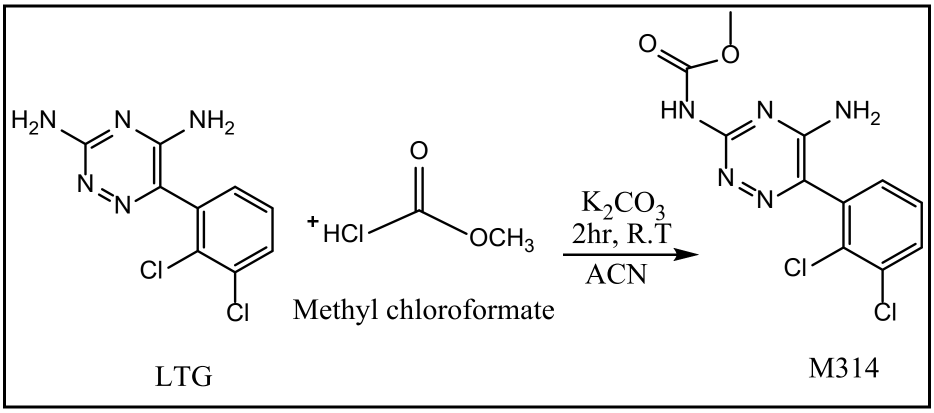

Scheme S5: The reaction scheme and conditions for the synthesis of the M314.

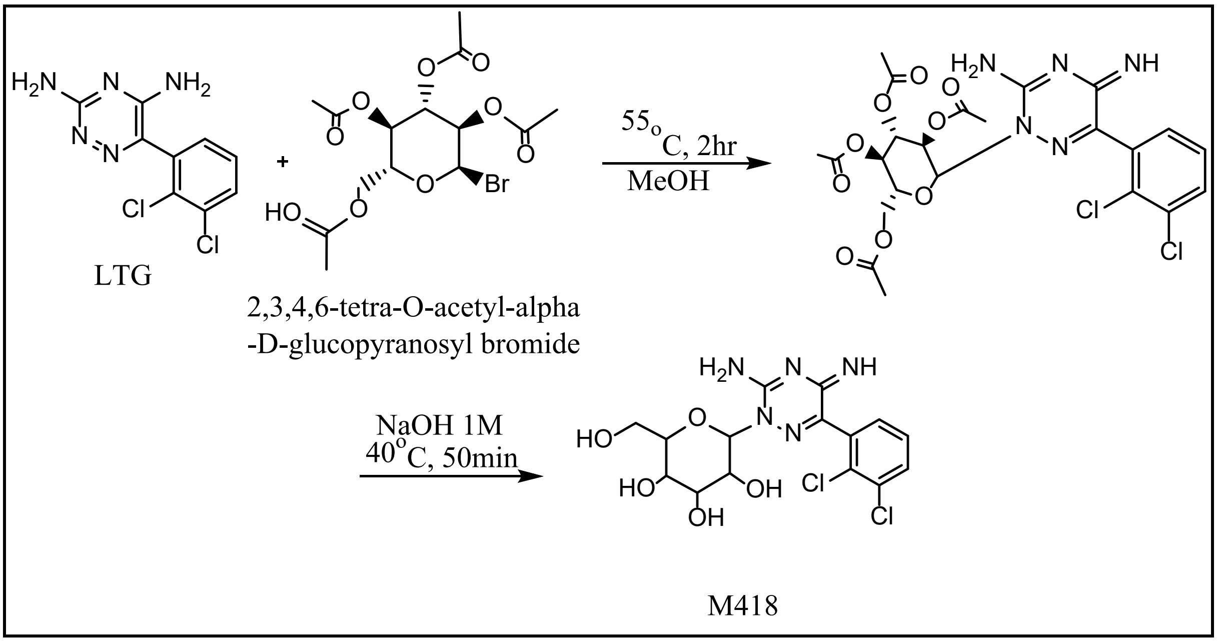

Scheme S6: The reaction scheme and conditions for the synthesis of the M418.

IDENTIFICATION OF LTG METABOLITES

Fragmentation patterns of lamotrigine metabolites after expose of cucumber plants ~~during 18 days~~, are presented. In the first section the MS/MS spectrum of identified metabolites are presented in comparison to a synthesized or a commercial standard (Figures S1-S7). In the following, the mass spectral information of the detected metabolites is presented (Figures S8-S16). All fragment ions colored in blue were observed in the fragmentation pattern of lamotrigine or lamotrigine metabolites. [1] The fragment ion at *m/z* 256.01 represents the mass of lamotrigine in its ionic state.

LTG-N2-Methyl

Mass spectrum of the M270 exhibited a quasi-molecular ion at *m/z* 270.0308 $[M+H]^+$ in positive-ion mode and the molecular formula was identified as $C_{10}H_{10}N_5Cl_2$ (LTG+CH_2) by high resolution MS (Table 1). The transformation probably suited to N-methylation of LTG. LTG-N_2-methyl was found as a minor metabolite in human urine as reported by Zonja et al. [2] The fragmentation patterns of the metabolite observed in the plants extract were compared to the fragmentation pattern of the commercially available standard, LTG-N_2-methyl. The comparison between the fragmentation patterns (Figure S1D vs. Figure S1E) and retention times (Figure S1A vs. Figure S1B) were executed and revealed a good match between the commercially standard and the M270, which was observed in the plant extract. Therefore, the confidence level is 1.

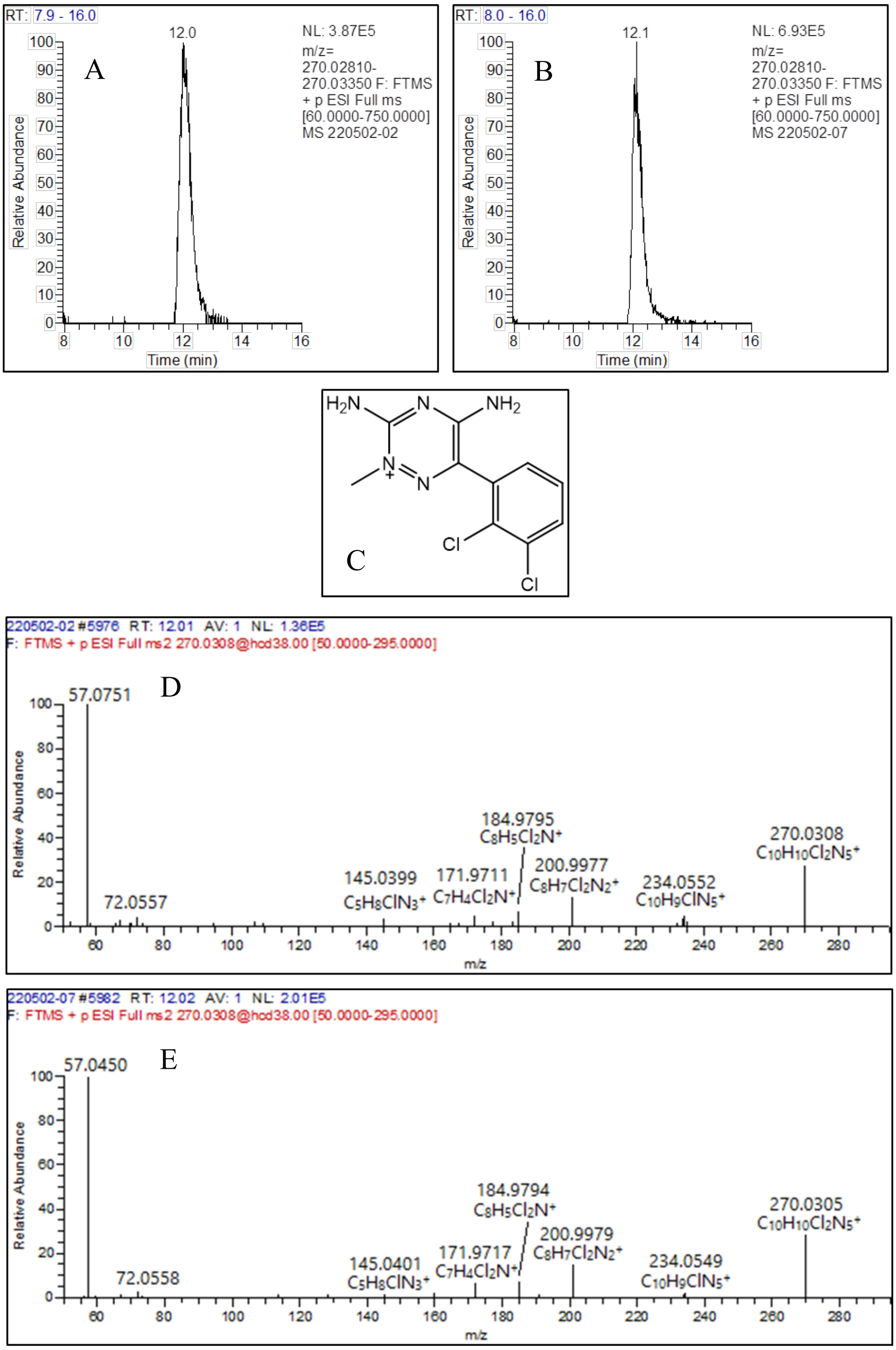

Figure S1. Identification of LTG-N_2-methyl. Full-MS chromatograms of the metabolite in plants extract, r.t = 12.0 min (A) and a commercial standard spiked into a control plants extract (B). A molecular structure (C) the ESI-MS/MS spectrum of LTG-N_2-methyl, in plants extract (D) and the ESI-MS/MS spectrum of the commercial standard (E), both at a collision energy of 38 eV.

M271

Mass spectrum of the M271 exhibited a quasi-molecular ion at *m/z* 271.0146 $[M+H]^+$ in positive-ion mode and the molecular formula was identified as $C_{10}H_9ON_4Cl_2$ (LTG-NH_2+OCH_3) by high resolution MS (Table 1). This modification could be suited to N-methylation of OXO-LTG. M271 was reported by Zonja et al. [2] in wastewater. As a commercial standard was not exist, we synthesized OXO-LTG-Me using OXO-LTG starting material and methyl iodide. The reaction scheme and conditions are provided in Scheme S2. The mass spectral data of M271 observed in the plants was compared to the synthesized standard. Both the retention time and the mass spectral fingerprint were matched (Figure S2). Full-MS chromatogram along with the ESI-MS/MS spectrum of M271 at a collision energy of 30 eV are depicted in Figure S2A and Figure S2D, respectively. The suggested structure (Figure S2C), Full-MS chromatogram along with the ESI-MS/MS spectrum of the synthesized standard spiked into the control sample at a collision energy of 30 eV are depicted in Figure S2B and Figure S2E, respectively. The confidence level is 3.

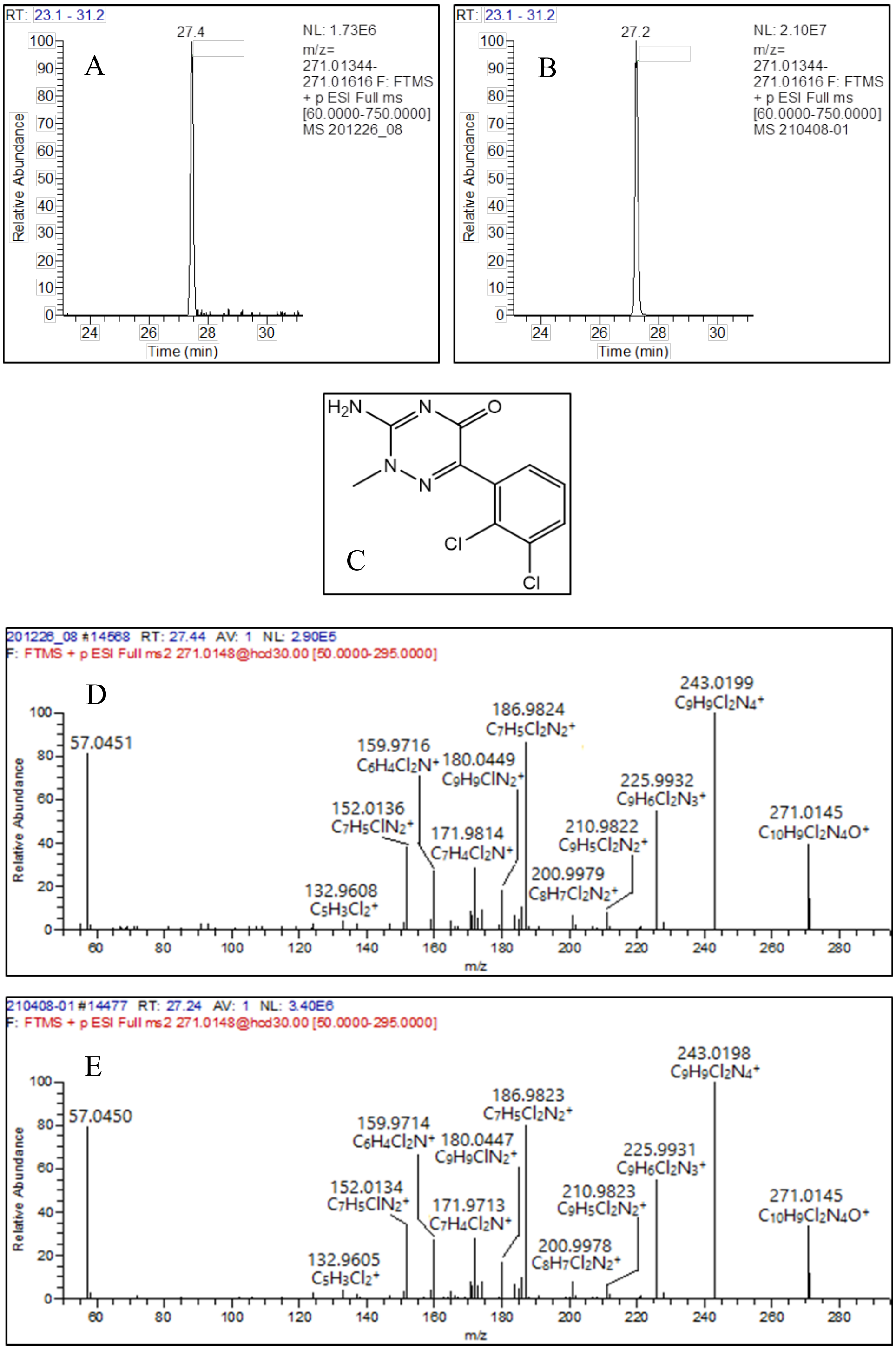

Figure S2. Identification of M271. Full-MS chromatograms of M271 in plants extract, r.t = 27.4 min (A) and spiked synthesized standard to a control plants extract (B). A suggested molecular structure (C), the ESI-MS/MS spectrum of M271, in plants extract (D) and the ESI-MS/MS spectrum of the synthesized proposed structure of M271 (E), both at a collision energy of 30 eV.

LTG-N2-oxide

Four chromatography peaks, eluted at different retention times with high-resolution mass spectrometry (HRMS) of *m/z* 272.0099, were identified as the oxidation products of LTG (LTG+O), as detailed in Table 1 and Figure S3A. The fragmentation patterns of the four isomers observed in the plants extract were compared to the fragmentation pattern of the commercially available standard, LTG-N_2-oxide. The ESI-MS/MS of peak observed at retention time 14.6 min (Figure S3D) and the ESI-MS/MS of the commercially available standard (Figure S3E) were completely matched. In addition, spiking of the commercially available standard LTG-N_2-oxide into the control samples was carried out revealing a chromatographic peak at the retention time of 14.6 min (Figure S3B). As the result, the structure of the metabolite was confirmed and the confidence level is 1.

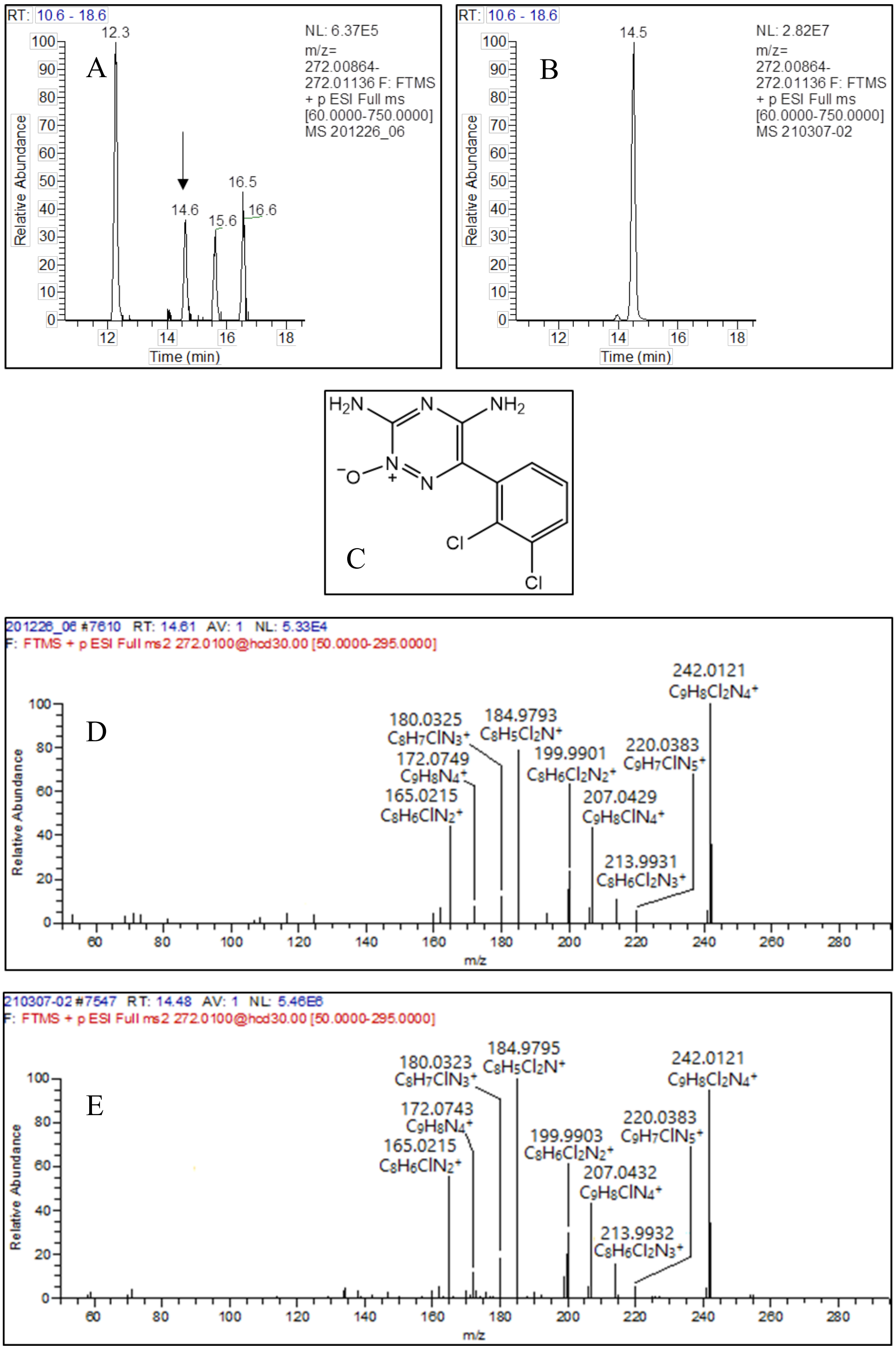

Figure S3. Identification of LTG-N_2-oxide. Full-MS chromatograms of this metabolite in plants extract, r.t = 14.6 min (A) and spiked synthesized standard to a control plants extract (B). A suggested molecular structure (C), the ESI-MS/MS spectrum in plants extract (D) and the ESI-MS/MS spectrum of the commercial standard of LTG-N_2-oxide (E), both at a collision energy of 30 eV.

M284

Mass spectrum of the M284 exhibited a quasi-molecular ion at *m/z* 284.0099 $[M+H]^+$ in positive-ion mode and the molecular formula was identified as $C_{10}H_8ON_5Cl_2$ (LTG+ CO) by high resolution MS. Full-MS chromatogram is depicted in Figure S4. The ESI-MS/MS spectra at a collision energy of 20 eV revealed a dominant product ion at *m/z* 256.0150 which corresponded to the loss of carbonyl (CO) from the precursor ion (data not shown). At a higher collision energy (e.g., 40 eV), in addition to ion at *m/z* 256.0149, other product ions representative of the LTG skeleton were also observed (Figure S4D). Thus, the CO moiety is suggested to conjugate to the N_2-LTG. Loss of carbonyl as a result of a cleavage at the C–N bond of the formamide group to generate the intact LTG is a common dissociation process [3] and a predicted structure was proposed (Figure S4C). To verify our assumption, the synthesis of LTG-formamide conjugate was produced under reflux of LTG with the formamide building block at basic conditions (see Scheme S3). As aforementioned for other metabolites we compared the fragmentation patterns of the peak observed in the plants extract (Figure S4D) to the fragmentation pattern of the synthesized standard spiked into the control sample (extract root of cucumber plants without exposure to LTG, Figure S4E). Ought to the identical MS/MS spectra match along with an identical retention time (r.t 16.8 min, Figure S4A versus Figure S4C) we deduced that our proposed structure is correct and the structure of M284 has been confirmed with confidence level 3. As aforementioned for other LTG-conjugates, the position of the carbonyl substituent is not determined.

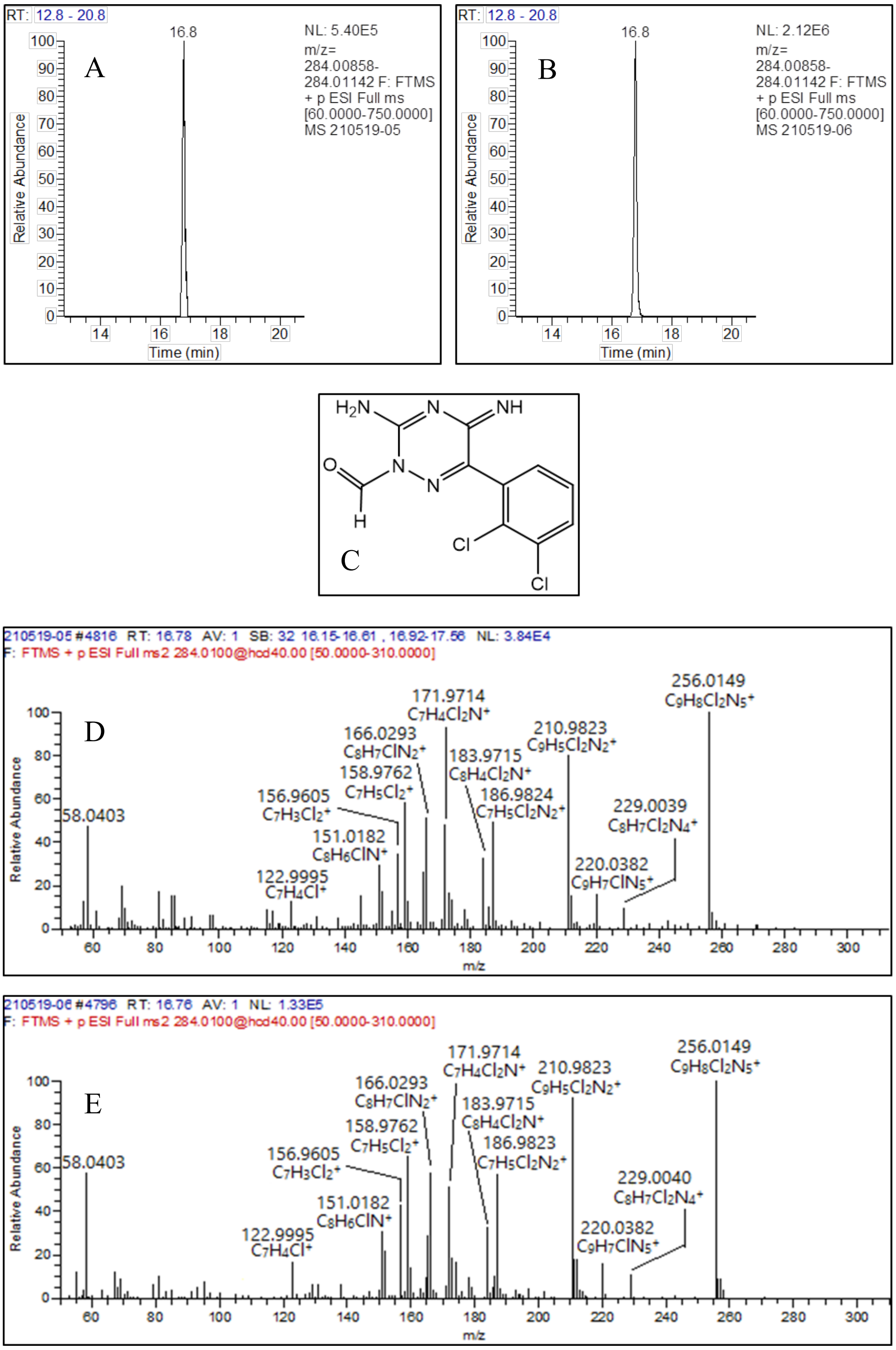

Figure S4. Identification of M284. Full-MS chromatograms of M284 in plants extract, r.t = 16.8 min (A) and spiked synthesized standard to a control plants extract (B). The suggested molecular structure (C), the ESI-MS/MS spectrum of M284, in plants extract (D) and the ESI-MS/MS spectrum of the synthesized proposed structure of M284 (E), both at a collision energy of 40 eV.

M312

Mass spectrum of the M312 exhibited a quasi-molecular ion at *m/z* 312.0411 $[M+H]^+$ in positive-ion mode and the molecular formula was identified as $C_{12}H_{12}ON_5Cl_2$ (LTG+ C_3H_4O, Table 1). The ESI-MS/MS spectra exhibited the formation of three product ions with higher masses than the product ion at *m/z* 256.0149 which was attributed to the intact protonated LTG. These three ions along with ion at *m/z* 256.0149 were the key role in structural elucidation process. The first was an ion at *m/z* 294.0305 which corresponded to loss of water from the *m/z* 312.0411 precursor ion; the second was an ion at *m/z* 268.0158 which corresponded to loss of C_2H_4O (epoxide or acetaldehyde) from the precursor ion; and the third ion was at *m/z* 266.0125. Loss of C_3H_4O from *m/z* 312.0411 precursor ion to produce product ion at *m/z* 256.0149, may be suited to the loss of acrylaldehyde as a result of a cleavage at the C–N bond of a potential amide group. As a result of the MS/MS data interpretation, three possible structures were raised as options. The first predicted structure of M312 was the formation of a propionamide moiety (a conjugation of propan-1-one group to one of the three amine nitrogen groups of LTG (Scheme S4A). Propionyl chloride was utilized as a reagent to react with LTG to produce the LTG-N_2-propan-1-one. The synthetic route is provided in the supporting information (Scheme S4A). The retention time and the MS/MS spectra of M312 in the plant's extract and the synthesized standard did not matched. Consequently, we predicted an alternative structure. The alternative candidate structure might have contained two groups: propenyl group attached to on nitrogen at the N_2-position and a hydroxyl group bound to the primary amine to produce a hydroxyl amine moiety (Scheme S4B). These two-step reactions involved the N-alkylation of LTG with allyl bromide followed by oxidation of the primary amine to form a hydroxyl amine. The proposed structure and the synthetic route are depicted in Scheme S4B. However, fragment ions ratio and retention times were not identical. The third proposed structure was the attachment of propene-1-ol to the LTG moiety which may tautomatizes (tautomer equilibrium) to form N-propionaldehyde moiety (a conjugation of propanal to one of the three amine nitrogen groups of LTG (Scheme S4C). Notably, the plausible structures of product ions at *m/z* 294.0303 and at *m/z* 268.0148 that were observed in the ESI-MS/MS of M312 were also detected in the ESI spectra of lamotrigine-N_2-glucurunide TP430 by Zonja et al., [2] and that strengthened our assumption on the proposed structure. We planned two-steps synthesis which included a substitution reaction between 2-(2-bromoethyl)-1,3-dioxalane (an acetal-protected bromopropione aldehyde) and the LTG, followed by a deprotected step of the acetal, utilizing acidic conditions. Comparison between the fragmentation patterns of the synthesized predicted compound (Figure S5E) and the suspected structure of M312 observed in the plants extract

sample (Figure S5D) revealed identical mass spectral fingerprint and retention time. The ESI-MS/MS spectra of both M312 and the synthesized predicted compound at a collision energy of 40 eV are displayed in Figure S5D and Figure S5E. The high intensities product ions at *m/z* 294.0302, 256.0147, 210.9818, 198.9820, 171.9713 and 81.0446 were presented at identical relative abundances. Therefore, the confidence level is 3, since the position of the substituent was not unequivocal.

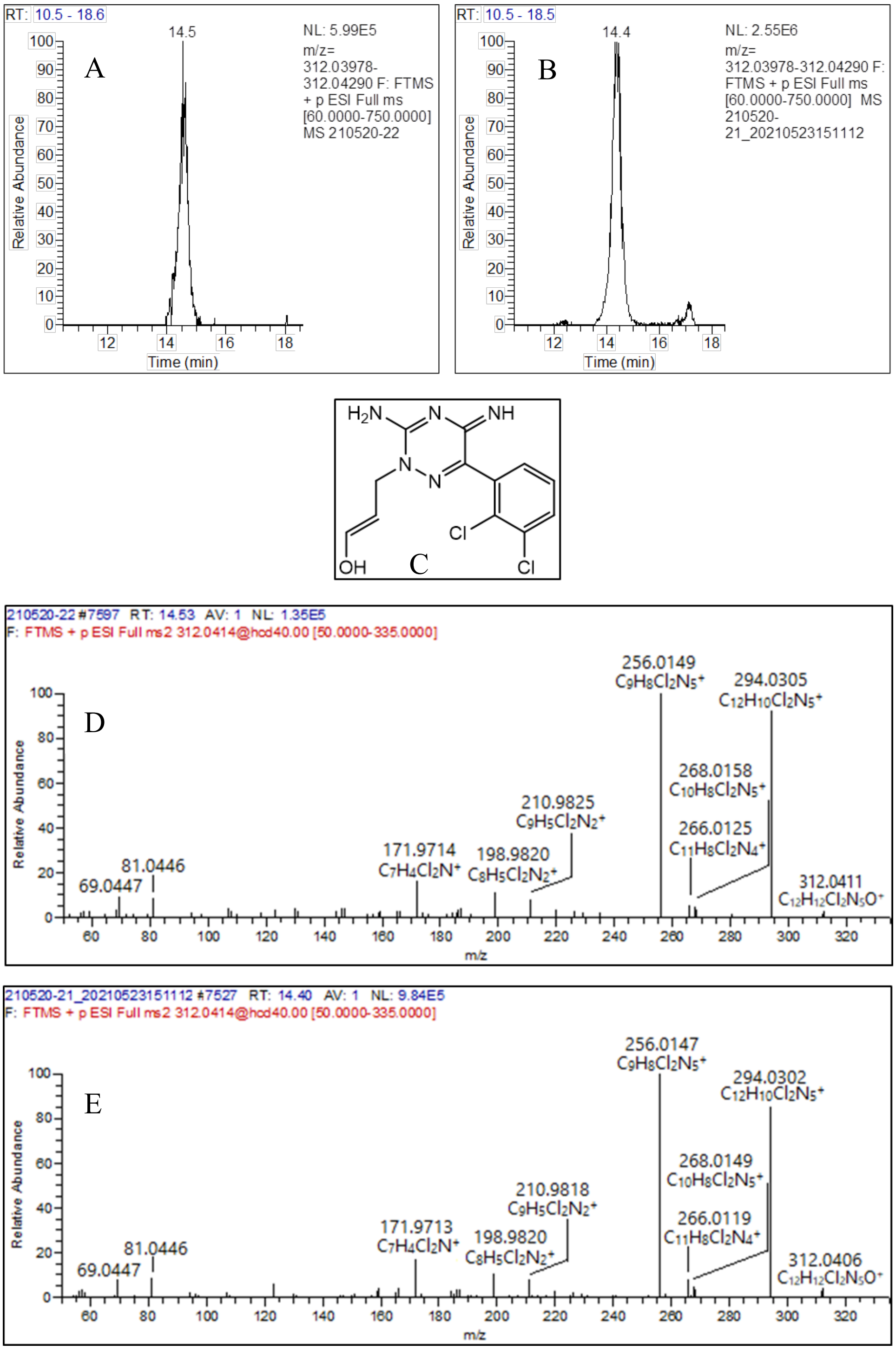

Figure S5. Identification of M312. Full-MS chromatograms of M312 in plants extract, r.t = 14.5 min (A) and spiked synthesized standard to a control plants extract (B). The suggested molecular structure (C), the ESI-MS/MS spectrum of M312, in plants extract (D) and the ESI-MS/MS spectrum of the synthesized proposed structure of M312 (E), both at a collision energy of 40 eV.

M314

Mass spectrum of the M314 exhibited a quasi-molecular ion at *m/z* 314.0206 $[M+H]^+$ in positive-ion mode and the molecular formula was identified as $C_{11}H_{10}O_2N_5Cl_2$ (LTG+$C_2H_2O_2$,, Table 1) by high resolution MS. Full-MS chromatogram along with the ESI-MS/MS spectrum of M314 at a collision energy of 40 eV are shown in Figures S6A and S6D, respectively. Although this molecular modification was reported in our previous work by white-rot fungus [4], its molecular structure is elucidated herein for the first time. Careful interpretation of the mass spectral information observed from the ESI-MS/MS spectra, revealed several product ions representative of the LTG skeleton (fragment ions that observed either in the ESI-MS/MS spectra of LTG or in its other reported conjugates). [1] This support strengthens our assumption that this metabolite is an LTG conjugate. A high intensity product ion at *m/z* 281.9942, was observed at a collision energy of 40 eV. This product ion was attributed to the loss of a methanol from *m/z* 314.0206 precursor ion. Since we assumed that one group is a methoxy group (OCH_3), the other group should be CO (a complementary to LTG+$C_2H_2O_2$). Carbamate groups are most likely to dissociate at the C–O bond with the charge remaining on the carbonyl group. [3] Protonation occurs at the ether oxygen of the carbamate group, followed by neutral loss of methanol. Therefore, our predicted structure of M314 was the conjugation of a methoxycarbonyl group to one of the three amine nitrogen groups of LTG (Figure S6C). The identification confidence levels of metabolites, based on exact mass measurement and MS/MS fragmentation data as aforementioned for M314, is only at level 3 as documented by Schymansky et al. [5]

Since there are still only limited MS/MS spectra in the public databases and the elucidation of M314 structure has not been published in literature. Moreover, a commercially available standard of M314 is not exist, therefore, a home-made synthesis was executed. For the synthesis of M314, methyl chloroformate was selected as a reagent to react with LTG. The detailed synthetic route is provided in the experimental section (Scheme S5). LC-HRMS analysis of peaks generated under reaction conditions, revealed the formation of three main products, two were probably attributed to the mono-substituted LTG and one was attributed to the di-substituted LTG. The formation of the two mono-substituted isomers products was observed due to the presence of more than one nucleophilic site in the molecule. We compared the fragmentation patterns of the two mono-substituted isomeric peaks which observed in the reaction mixture (Figure S6E), to the fragmentation pattern of the peak in the plants extract. The ESI-MS/MS spectra of the precursor ion at *m/z* 314.020 of both mono-substituted isomers contained an informative product ion at 281 (loss of methanol), as expected, however, the mass

spectral fingerprint of only one isomer was completely identical (masses and their relative abundances, Figure S6E). In addition, the reaction mixture, which contained the two isomeric compounds, was spiked into a control plants extract and the retention times found to be identical.

Ought to the retention time and MS/MS spectra match, we proved that the structure of the M314 in the plant extract and the synthesis candidate is identical and therefore, the structure of M314 has been confirmed with a higher level of confidence. Notably, further investigation is underway to determine the exact position of the substituted nitrogen. Therefore, the identification level is 3.

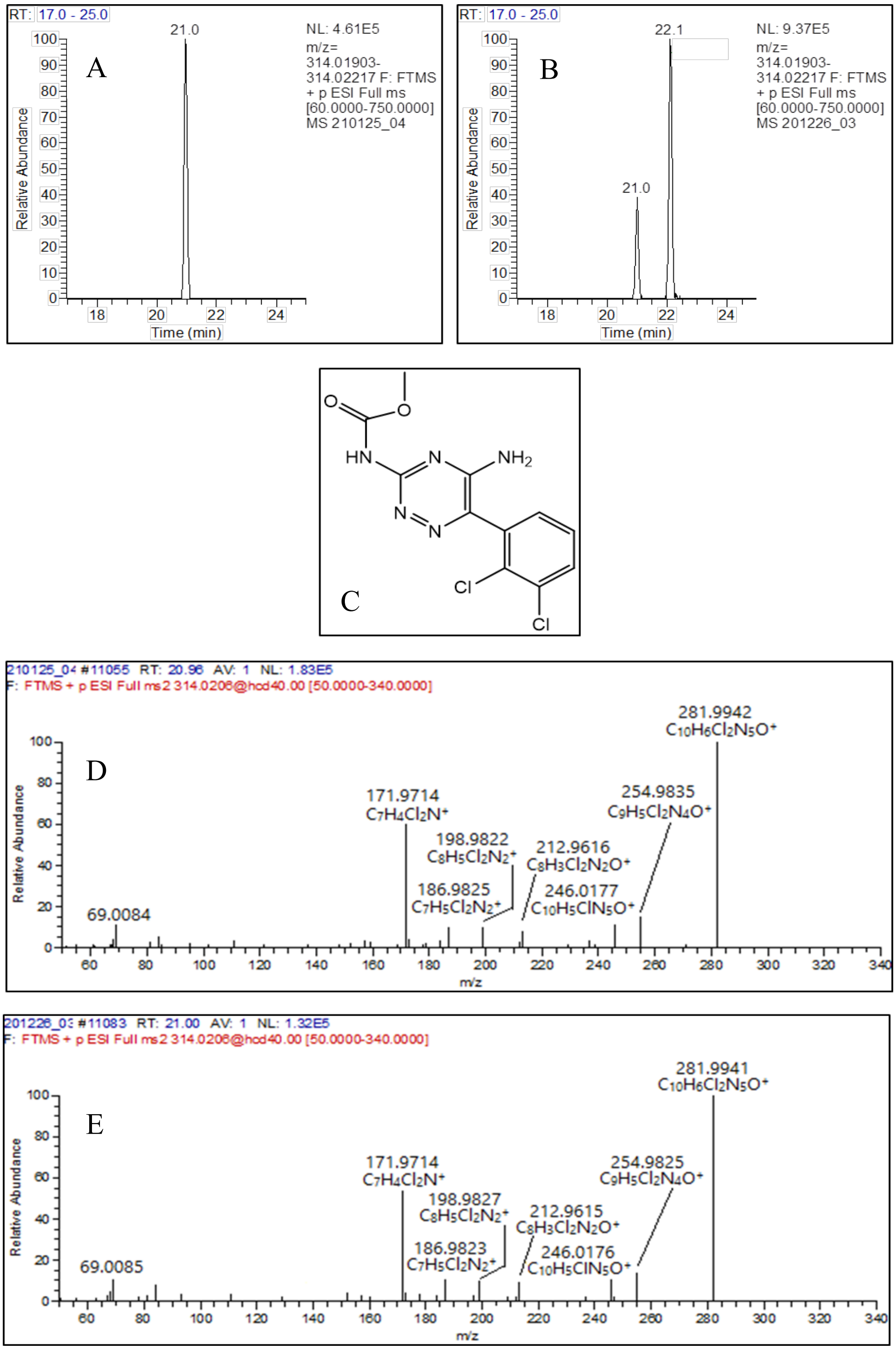

Figure S6. Identification of M314. Full-MS chromatograms of M314 in plants extract, r.t = 21.0 min (A) and spiked synthesized standard to a control plants extract (B). The suggested molecular structure (C), the ESI-MS/MS spectrum of M314, in plants extract (D) and the ESI-MS/MS spectrum of the synthesized proposed structure of M314 (E), both at a collision energy of 40 eV.

M418

Mass spectrum of the M418 exhibited a quasi-molecular ion at *m/z* 418.0675 $[M+H]^+$ in positive-ion mode and the molecular formula was identified as $C_{15}H_{18}O_5N_5Cl_2$ (LTG+ $C_6H_{10}O_5$, Table 1) by high resolution MS. The ESI-MS/MS spectra at collision energies (10-50 eV) revealed a single dominant product ion at *m/z* 256.0148 representative of the intact LTG (data not shown). Full-MS chromatogram along with the ESI-MS/MS spectrum of M418 at a collision energy of 10 eV are depicted in Figures S7A and S7D, respectively. The only information that can be deduced from the ESI-MS/MS spectrum is the difference in mass between the precursor ion at *m/z* 418.0676 and the product ion at *m/z* 256.0148. According to literature, N-glucuronidation of LTG pathway is the major route of metabolism in humans as N_2-glucuronide and N_5-glucuronide. [1] Although N-glucuronidation metabolite *m/z* 432 was not detected in the plant's extract, a quasi-molecular ion at *m/z* 418.0675 which might be suitable to the conjugation of glucose to the LTG skeleton was detected. Therefore, our predicted structure of M418 was LTG-N-glucosidation (Figure S7C). Notably, inspection of the LTG structure reveals three possible amino-imino tautomers. The fact that The N_2-position is favorable as N_2-methyl, N_2-oxide and N_2-glucuronide metabolites in human may indicate that the basicity the N_2-position is high. Therefore, we proposed the LTG-N_2-glucosidation structure.

Since glucosidation of LTG has not been reported in literature, and in addition, there is no a commercially available standard of M418, a home-made synthesis was carried out. 2, 3, 4, 6 tetra O-acetyl glucopyranosyl bromide was selected as a building block to react with LTG to produce the protected N-glucosidation metabolite M418. Further step was the hydrolysis of the acetyl protecting groups, without isolation of the intermediate, using aqueous sodium hydroxide solution. Detailed synthetic route and procedure are provided in the supporting information (Scheme S6) and in the experimental section, respectively. LC-HRMS analysis of a peak generated under synthetic reaction conditions revealed the presence of the exact molecular mass suitable to that of the N-glucosidation product, probably attributed to the LTG-N2-glucosidation. To ascertain our predicted structure, retention times (Figure S7A versus Figure S7B) and the fragmentation patterns (Figure S7D versus S7E) were compared. The retention time and the mass spectral fingerprint of M418 and the synthesized standard were matched. Although the mass spectral match was based on the presence of a precursor ion at *m/z* 418.0675 and a single product ion at *m/z* 256.0148 with a relative intensity of 1~1, it is noteworthy that at collision energies above 50 eV, indicative product ions of the LTG-core were also observed (data not shown).

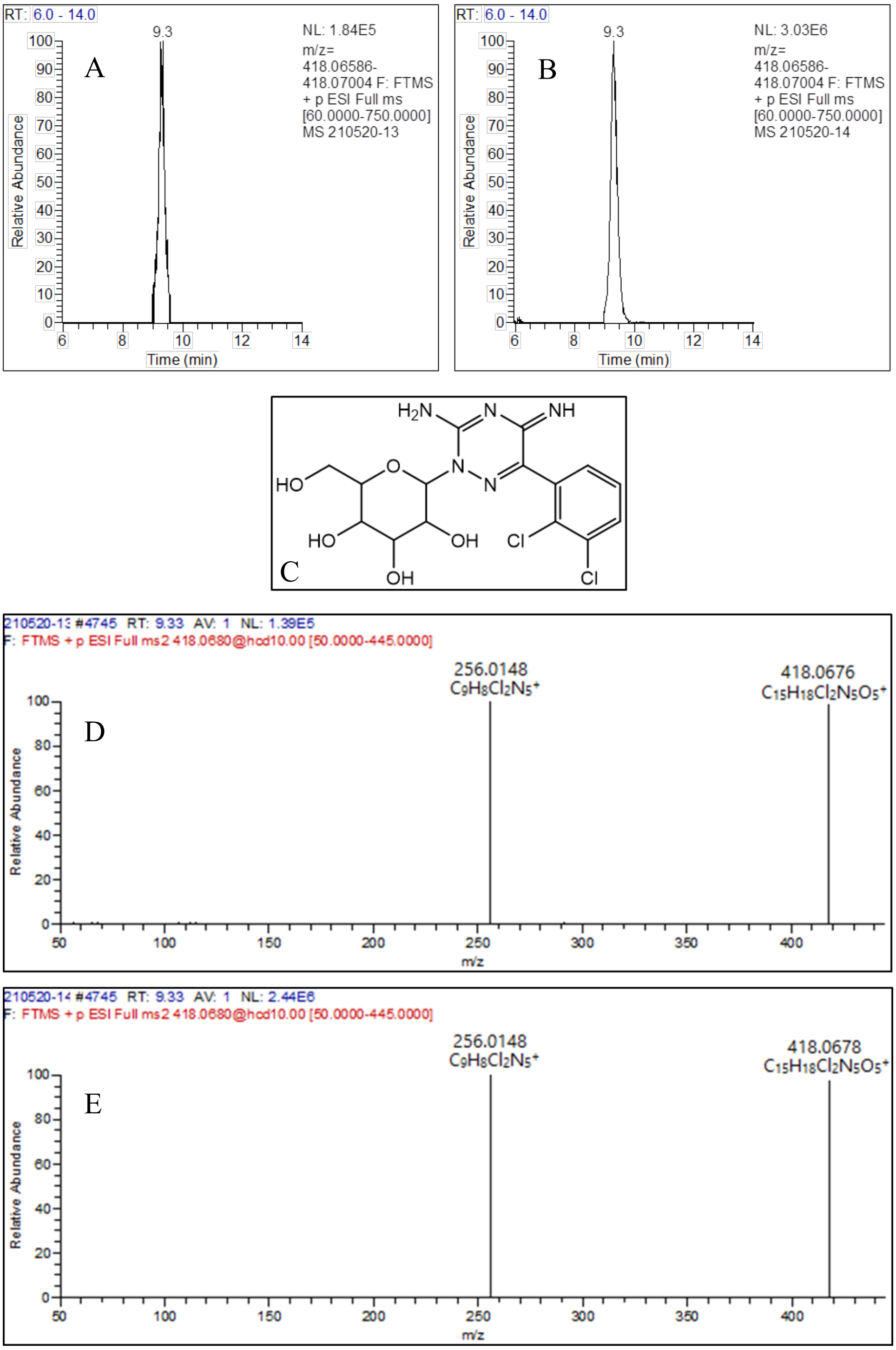

Figure S7. Identification of M418. Full-MS chromatograms of M418 in plants extract, r.t = 9.3 min (A) and spiked synthesized standard into a control plants extract (B). The suggested molecular structure (C), the ESI-MS/MS spectrum of M418, in plants extract (D) and the ESI-MS/MS spectrum of the synthesized proposed structure of M418, both at a collision energy of 10 eV (E).

DETECTION OF OTHER LTG METABOLITES

M272

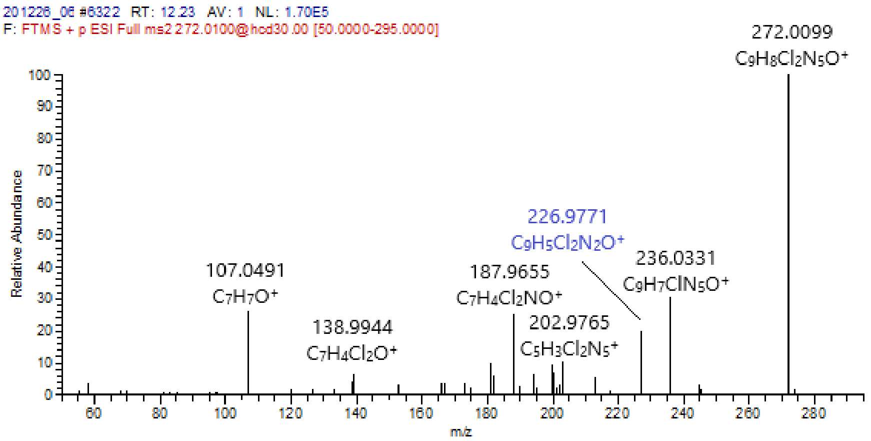

Figure S8. Detection of M272. ESI-MS/MS spectrum of M272 observed in roots extract. The collision energy and retention time were 30 eV and 12.2 min, respectively. The fragment ion at *m/z* 226.9771 – $C_9H_5Cl_2N_2O^+$ was observed in the fragmentation pattern of LTG-N_2-glucuronide.

M354

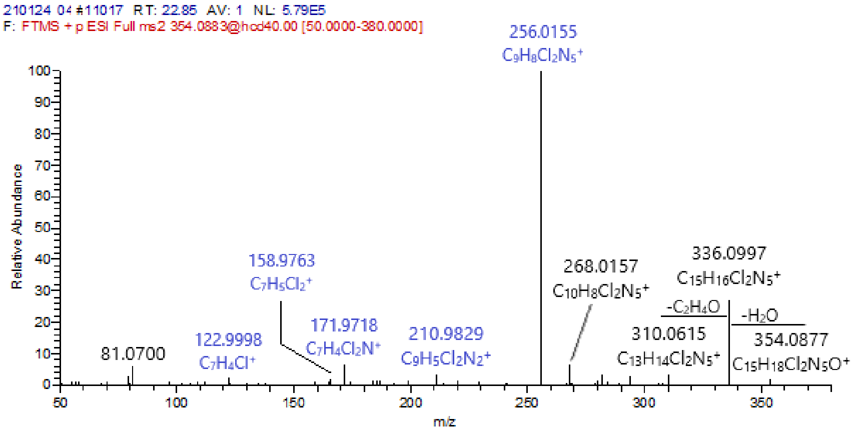

Figure S9. Detection of M354. ESI-MS/MS spectrum of M354 observed in roots extract. The collision energy and retention time were 40 eV and 22.8 min, respectively. The fragment ion at *m/z* 171.9712 - $C_7H_4Cl_2N^+$ was observed in the fragmentation pattern of LTG-N_2-glucuronide and the other fragment ions that colored in blue are attributed to lamotrigine core structure. At a higher collision energy, these ions were obtained at higher intensities and the fragmentation pattern was similar to the fragmentation pattern of lamotrigine.

M362

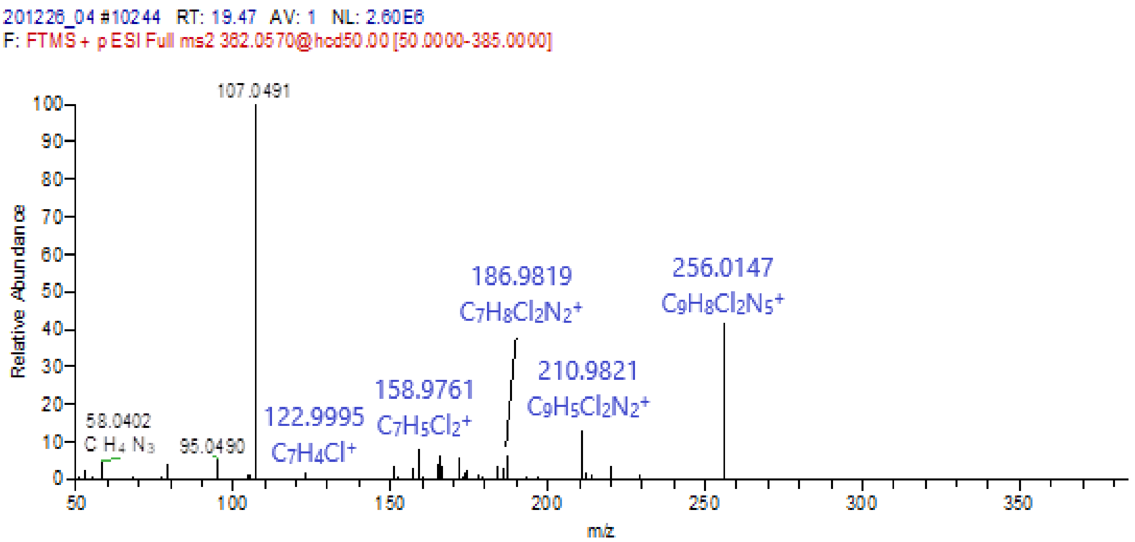

Figure S10. Detection of M362. ESI-MS/MS spectrum of M362 observed in roots extract. The collision energy and retention time were 50 eV and 19.5 min, respectively. All the fragment ions that colored in blue are attributed to the fragmentation pattern of lamotrigine.

M368

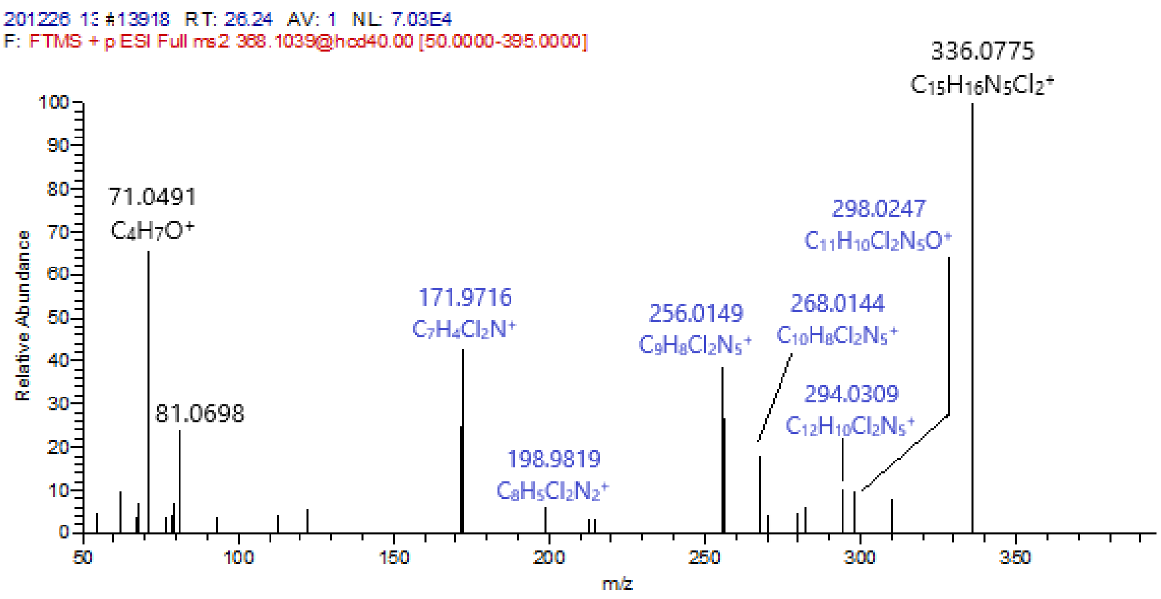

Figure S11. Detection of M368. ESI-MS/MS spectrum of M368 observed in roots extract. The collision energy and retention time were 40 eV and 26.2 min, respectively. The fragments ion colored in blue were observed in the MS/MS spectra of the LTG-N_2-glucuronide metabolite.

M370

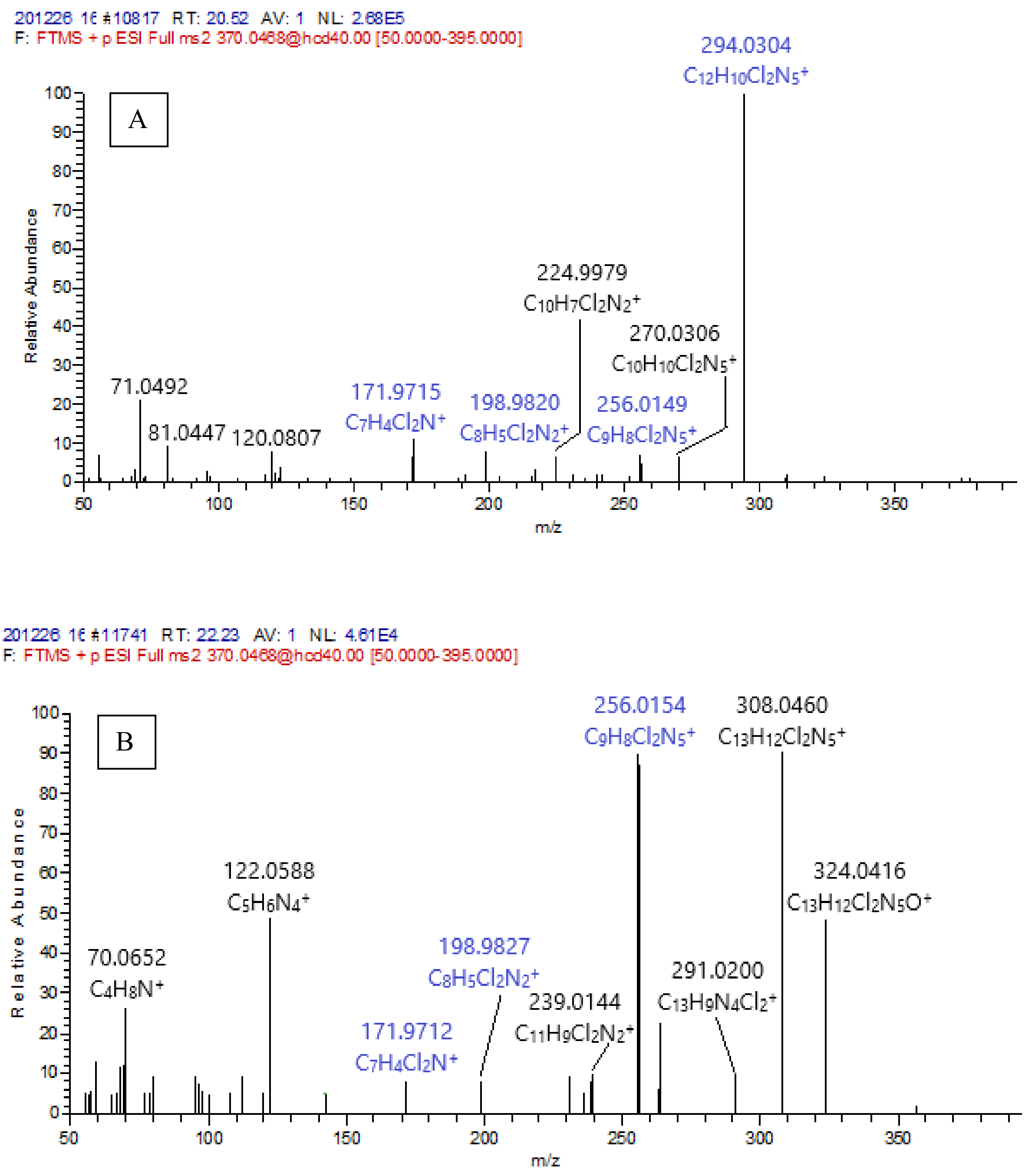

Figure S12. Detection of M370 of two isomers (A+B). ESI-MS/MS spectrum of M370 observed in roots extract. The collision energy and retention time for both isomers were 40 eV and 20.5 min (A) and 22.2 min (B), respectively. The fragment ions colored in blue were observed in the MS/MS spectra of the LTG-N_2-glucuronide metabolite.

M372

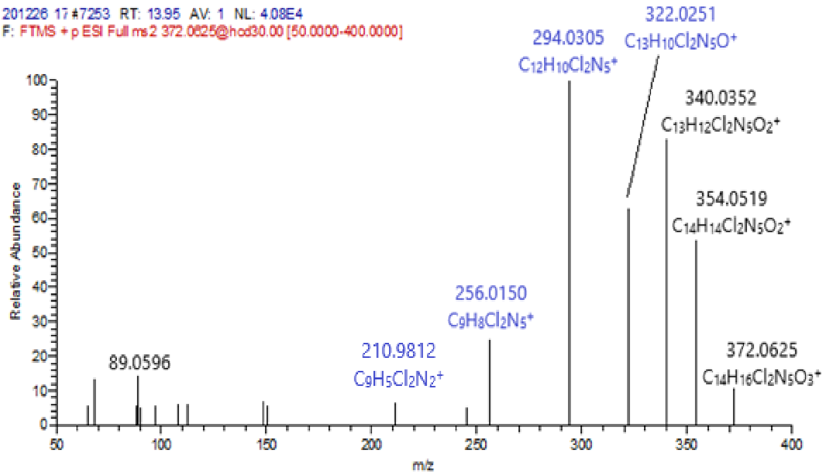

Figure S13. Detection of M372. ESI-MS/MS spectrum of M372 observed in roots extract. The collision energy and retention time were 30 eV and 13.9 min, respectively. The ions colored in blue are the same as those reported for the LTG-N2-glucurinide metabolite. At a higher collision energy, the fragment ions 198.9831 - $C_8H_5N_2Cl_2^+$ and 171.9714 - $C_7H_4Cl_2N^+$ were observed. There ions reported in the fragmentation patterns of lamotrigine and the LTG-N2-glucuronide metabolite, respectively.

M409

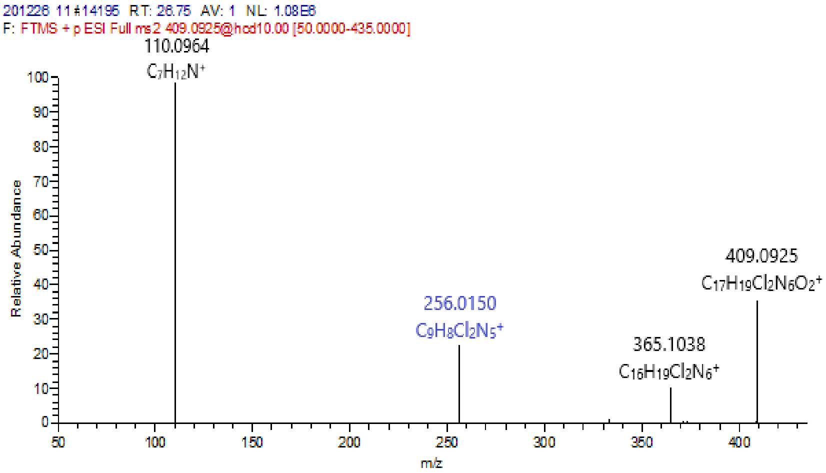

Figure S14. Detection of M409. ESI-MS/MS spectrum of M409 observed in roots extract. The collision energy and retention time were 10 eV and 26.7 min, respectively. The fragmentation patterns for both isomers were similar. Additionally, at high collision energies, the fragments ions at *m/z* 210.9825 and *m/z* 186.9828 were observed in the ESI-MS/MS of LTG-N2-glucuronide metabolite and lamotrigine, respectively.

M430

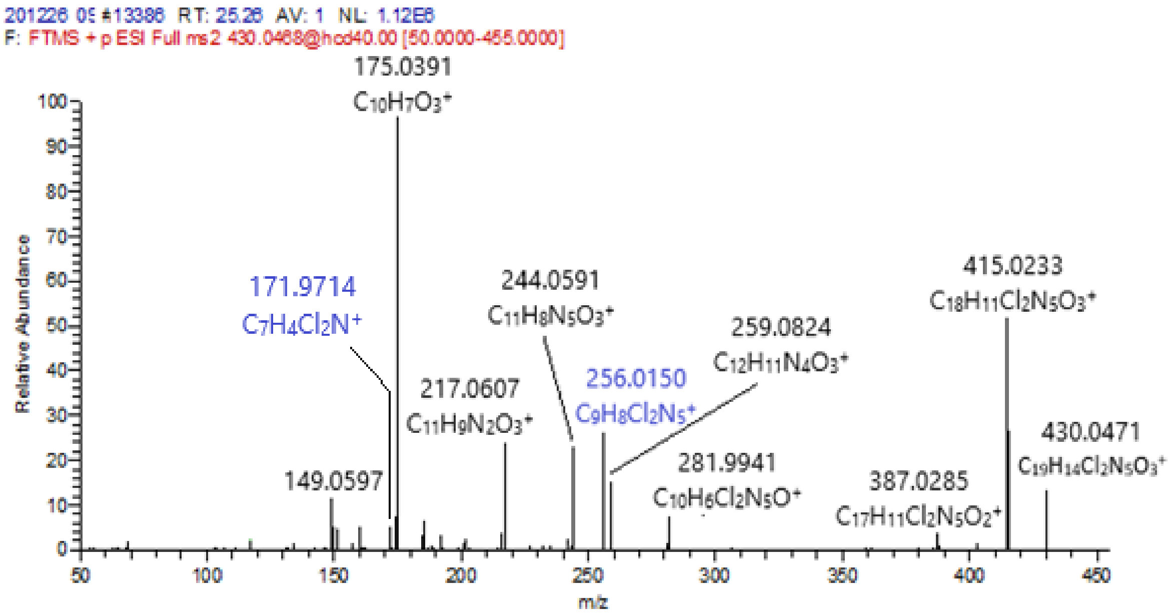

Figure S15. Detection of M430. ESI-MS/MS spectrum of M430 observed in roots extract. The collision energy and retention time were 40 eV and 25.3 min, respectively.

M468

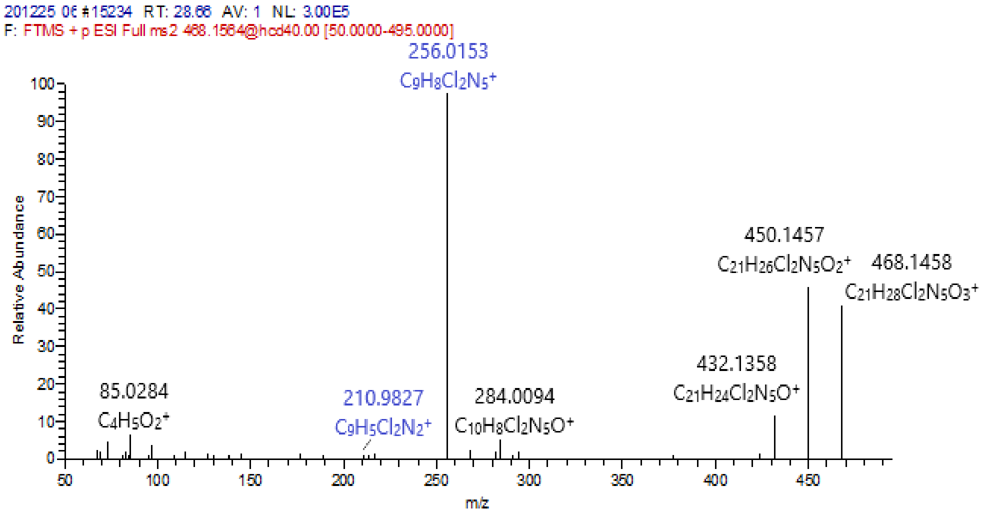

Figure S16. Detection of M468. ESI-MS/MS spectrum of M468 observed in roots extract. The collision energy and retention time were 40 eV and 28.7 min, respectively. At higher collision energy, additional fragment ions were observed such as *m/z* 186.9827 - $C_7H_5Cl_2N_2^+$ and *m/z* 158.9762 - $C_7H_5Cl_2^+$ which are identical to the fragment ions of lamotrigine core structure. The fragment ion at *m/z* 171.9712 - $C_7H_4Cl_2N^+$ was observed in the MS/MS spectra of the LTG-N_2-glucuronide metabolite.

DISTRIBUTION PROFILES OF LTG METABOLITES

For all figures (S17-S30), Y axis shows average data (peak area normalized to peak area of LTG-$^{13}C_3$) of 5 replicates, bars represent standard errors. X axis show days of exposure. Data shown for roots, stem and leaves for metabolites with confidence levels 3 and 4.

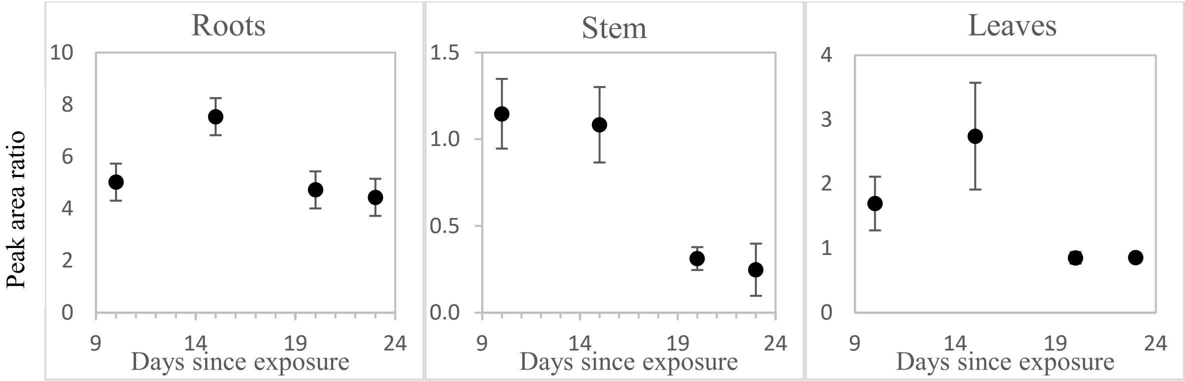

Figure S17. Distribution profile of M271.

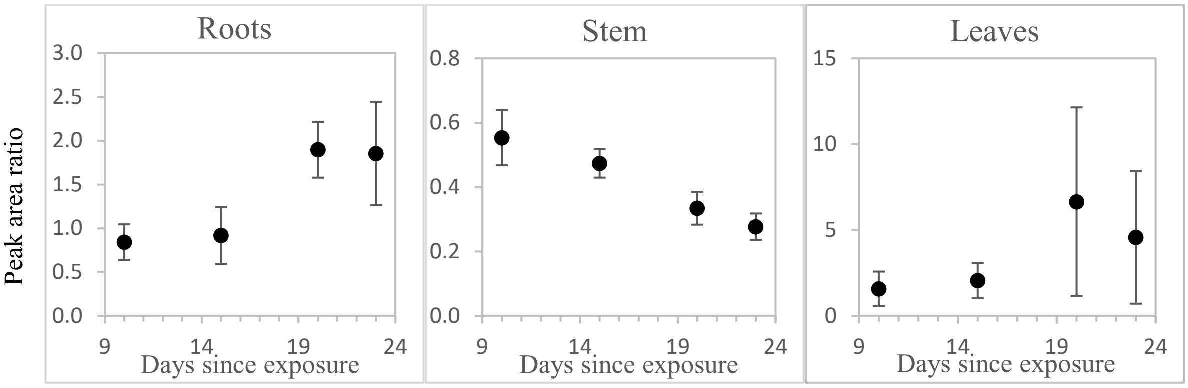

Figure S18. Distribution profile of M272.

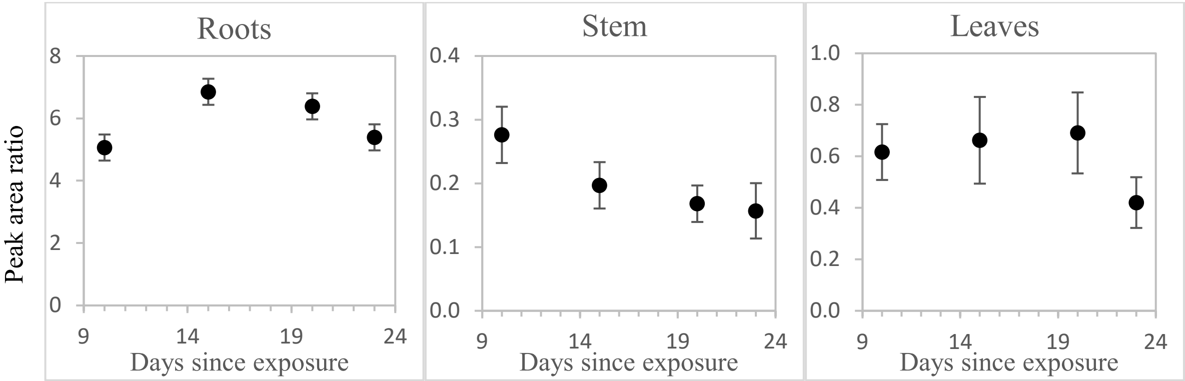

Figure S19. Distribution profile of M284.

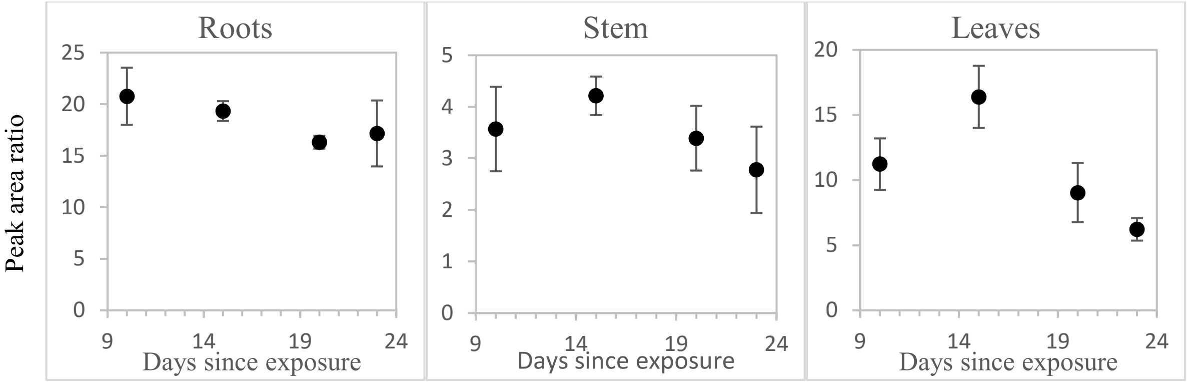

Figure S20. Distribution profile of M312.

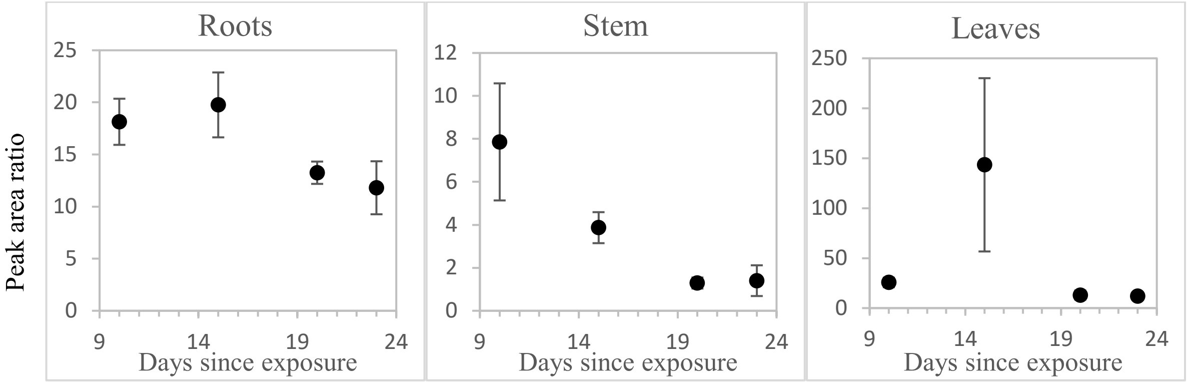

Figure S21. Distribution profile of M314.

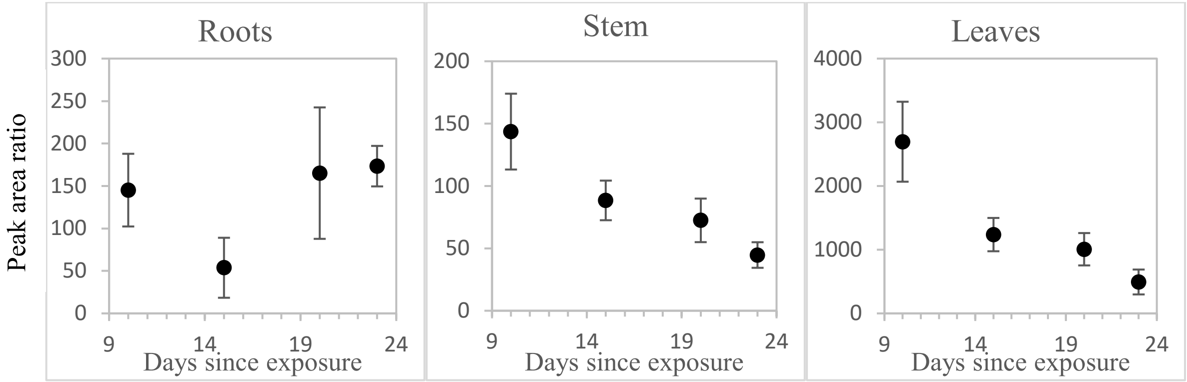

Figure S22. Distribution profile of M354.

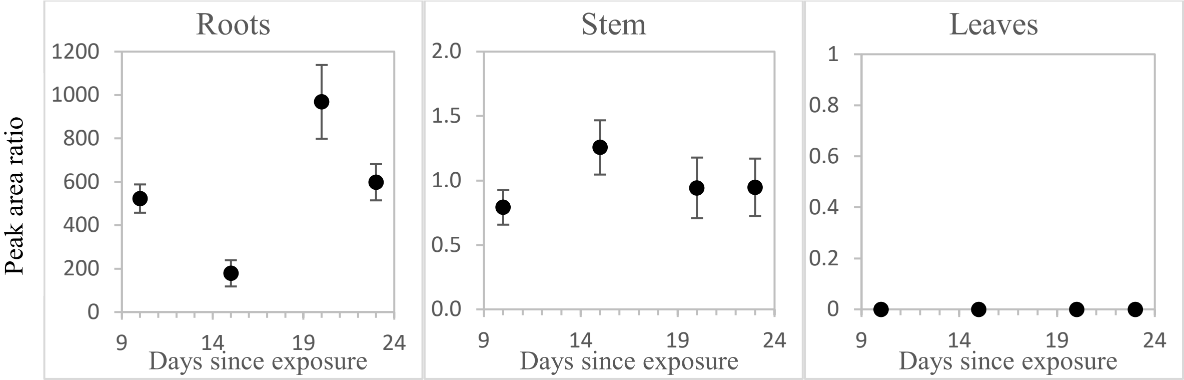

Figure S23. Distribution profile of M362.

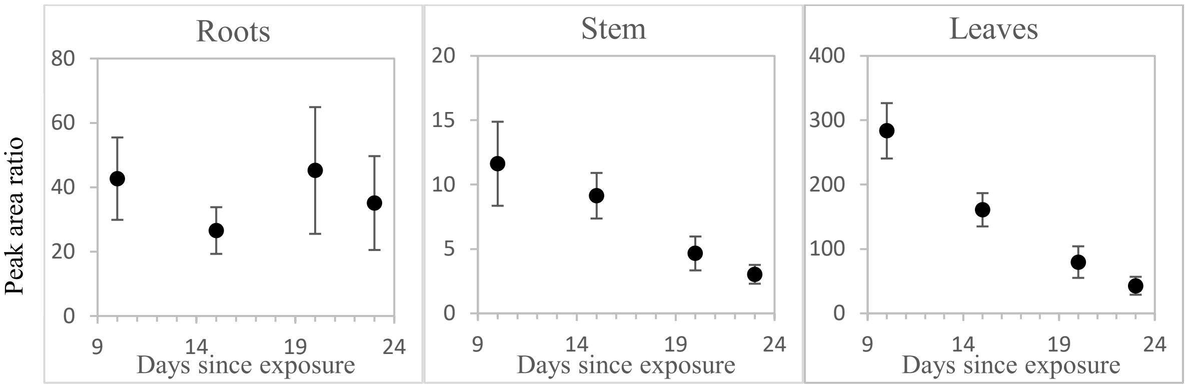

Figure S24. Distribution profile of M368.

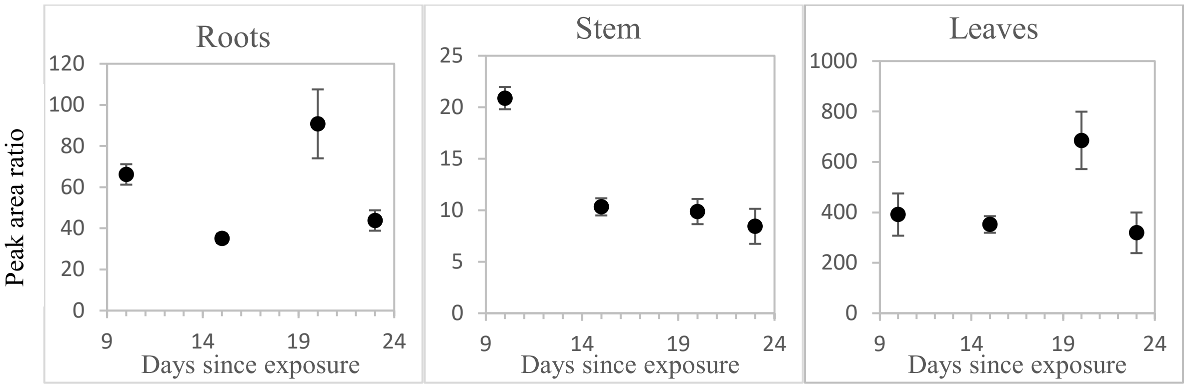

Figure S25. Distribution profile of M370.

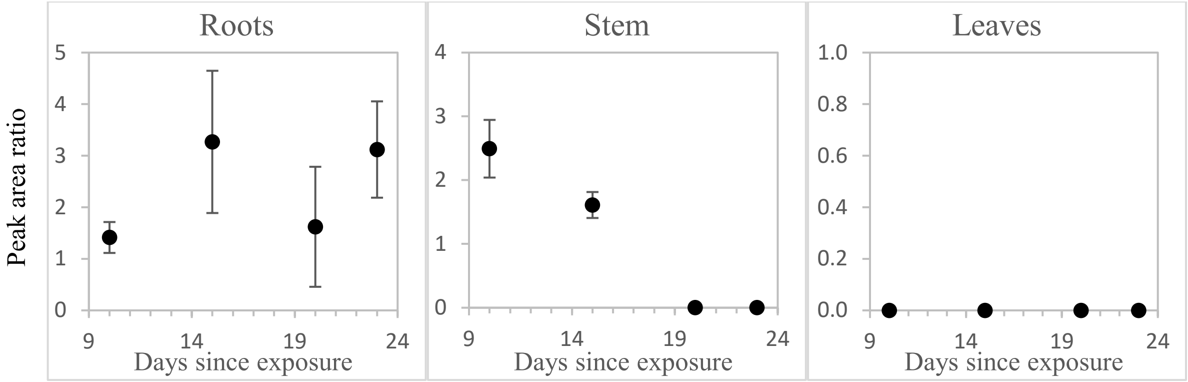

Figure S26. Distribution profile of M372.

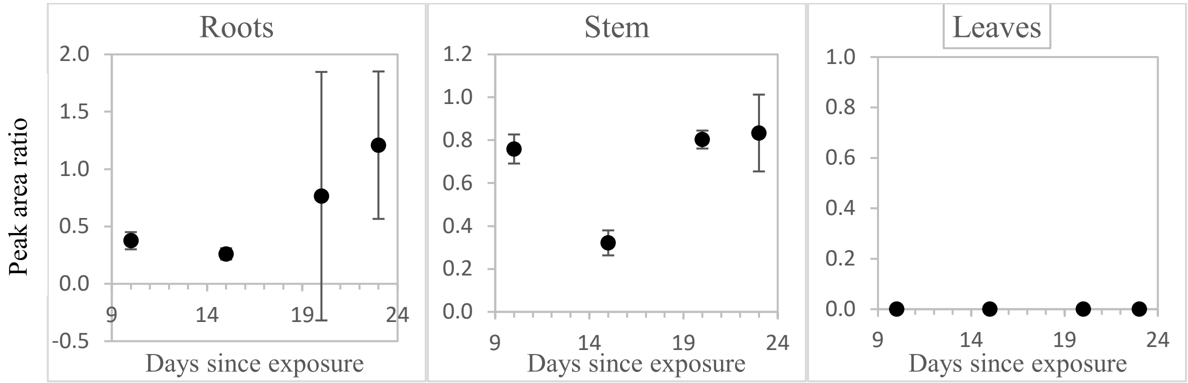

Figure S27. Distribution profile of M409.

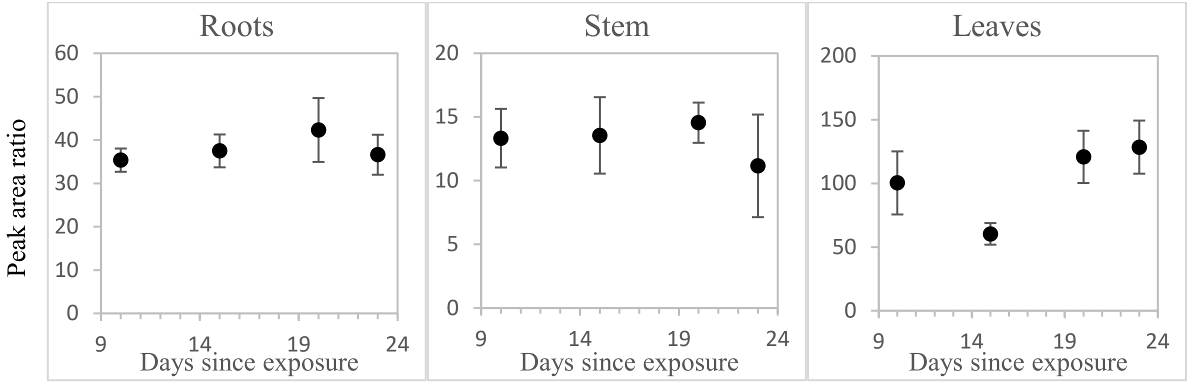

Figure S28. Distribution profile of M418.

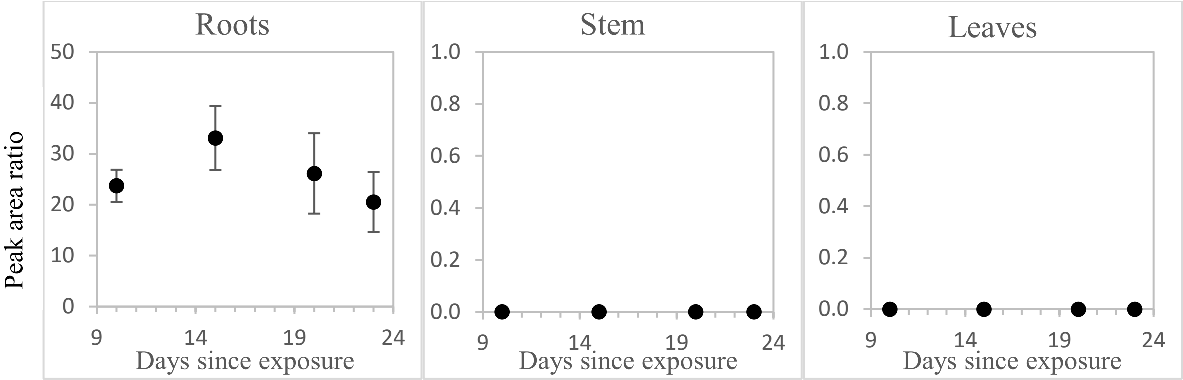

Figure S29. Distribution profile of M430.

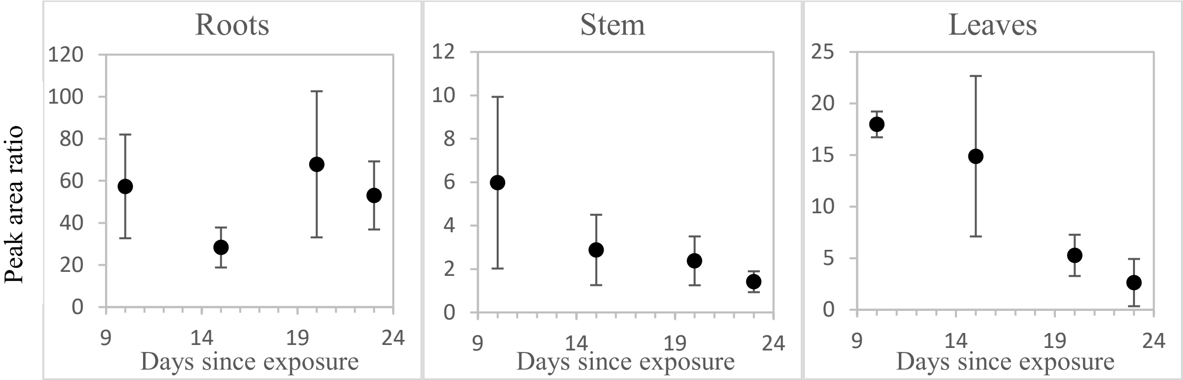

Figure S30. Distribution profile of M468.

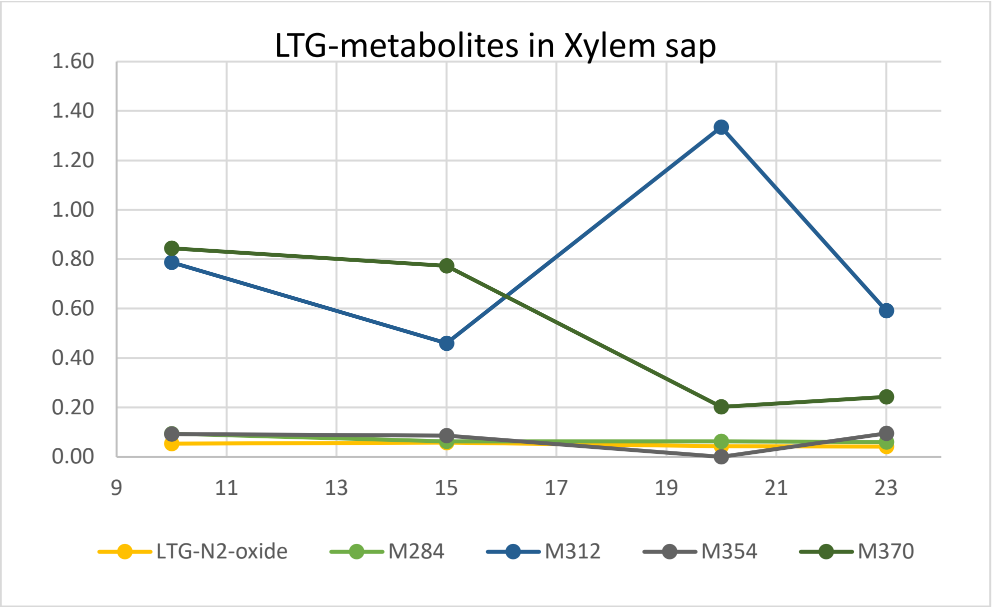

Figure S31. LTG-metabolites that detected in xylem sap during the exposure period.

References

1. Zonja, B.; Pérez, S.; Barceló, D. Human metabolite lamotrigine-N2-glucuronide is the principal source of lamotrigine-derived compounds in wastewater treatment plants and surface water. *Environ. Sci. Technol.* **2016**, 50, 154−164.
2. Zonja, B.; Delgado, A.; Abad, J. L.; Pérez, S.; Barceló, D. Abiotic amidine and guanidine hydrolysis of lamotrigine-N2-glucuronide and related compounds in wastewater: The role of pH and N2-substitution on reaction kinetics. *Water Res.* **2016**, 100, 466–475.
3. Weissberg, A.; Dagan, S. Interpretation of ESI(+)-MS-MS spectra-towards the identification of "unknowns". *Int. J. Mass Spectrom.* **2011**, 299 (2-3), 158–168.
4. Chefetz, B.; Marom, R.; Salton, O.; Oliferovsky, M.; Mordehay, V.; Ben-Ari, J.; Hadar, Y. Transformation of lamotrigine by white-rot fungus Pleurotus ostreatus. *Environ. Pollut.* **2019**, 250, 546–553.
5. Schymanski, E. L.; Jeon, J.; Gulde, R.; Fenner, K.; Ruff, M.; Singer, H. P.; Hollender, J. Identifying small molecules via high resolution mass spectrometry: Communicating confidence. *Environ. Sci. Technol.* **2014**, 48, 2097–2098.
